# LPMO-Catalyzed Oxidation of Cellulosic Fibers with Controlled Addition of a Reductant and H_2_O_2_

**DOI:** 10.1021/acssuschemeng.4c06802

**Published:** 2024-12-30

**Authors:** Kaisa Marjamaa, Jenni Rahikainen, Fredrik G. Støpamo, Irina Sulaeva, Waltteri Hosia, Natalia Maiorova, Alistair W. T. King, Antje Potthast, Kristiina Kruus, Vincent G. H. Eijsink, Anikó Várnai

**Affiliations:** † VTT Technical Research Centre of Finland, P.O. Box 1000, Espoo, FI-02044 VTT, Finland; ‡ Norwegian University of Life Sciences (NMBU), Faculty of Chemistry, Biotechnology and Food Science, Chr. Magnus Falsens vei 18, Ås 1433, Norway; § University of Natural Resources and Life Sciences (BOKU), Konrad Lorenz-Straße 24, Tulln an der Donau A-3430, Austria; ∥ Aalto University, P.O. Box 16100, Espoo, 00076 Aalto, Finland

**Keywords:** cellulose, fiber engineering, LPMO, controlled oxidation, hydrogen peroxide, SEC-MALS

## Abstract

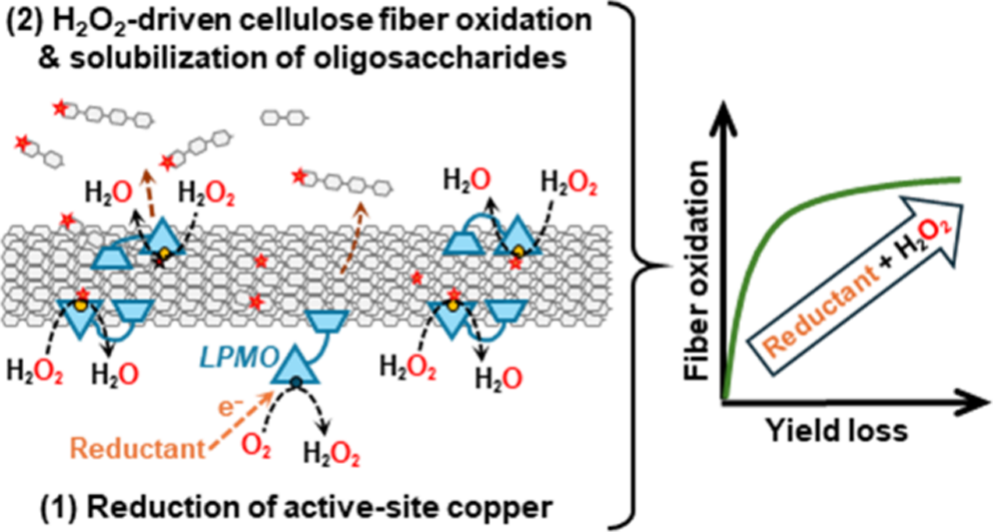

Cellulose-derived biomaterials offer a sustainable and versatile platform for various applications. Enzymatic engineering of these fibers, particularly using lytic polysaccharide monooxygenases (LPMOs), shows promise due to the ability to introduce functional groups onto cellulose surfaces, potentially enabling further functionalization. However, harnessing LPMOs for fiber engineering remains challenging, partly because controlling the enzymatic reaction is difficult and partly because limited information is available about how LPMOs modify the fibers. In this study, we explored controlling LPMO-mediated fiber oxidation by sequentially adding a reductant (gallic acid, GA) and H_2_O_2_, using three different carbohydrate-binding module (CBM)-containing LPMOs. An in-depth analysis of the soluble products and the *M*_n_, *M*_w_, and carbonyl content in the fiber fraction indicates that fiber oxidation can indeed be controlled by adjusting the amount of GA and H_2_O_2_ added to the reaction. In particular, at lower overall dosages of GA and H_2_O_2_, corresponding to low oxidation levels, fiber oxidation occurs rapidly with almost no release of soluble oxidized products. Conversely, at higher dosages, fiber oxidation levels off, while oxidized oligosaccharides continue to be released and the fibers are eroded. Importantly, next to demonstrating controlled fiber oxidation, this study shows that different cellulose-active LPMOs modify the fibers in different manners.

## Abstract 2

Controlled enzymatic oxidation of cellulose fibers by LPMOs offers a sustainable and targeted alternative to conventional chemical functionalization processes.

## Introduction

Cellulosic fibers are widely used in materials ranging from paper and textile to cellulose derivatives and nanomaterials.^1^ Cellulose is characterized by dense intra- and intermolecular hydrogen bond networks, which contribute to its rigid, partially crystalline structure. In plant fibers, cellulose molecules are organized into nanoscale elementary fibrils and further into microfibrils, which are organized in the copolymeric plant cell wall with excellent mechanical strength. As a consequence, processing of cellulose-rich plant fibers tends to be energy- and chemical-intensive.^2,3^

Enzymes offer a more sustainable means to reduce energy and chemical consumption in the processing of fibers for various products.^4−7^ Enzymes that act on carbohydrates are classified in the Carbohydrate-Active enZymes (CAZy) database (http://www.cazy.org/).^8^ The most commonly applied enzymes in cellulose fiber processing are glycoside hydrolases (GHs), including cellulases, xylanases, and mannanases. These enzymes hydrolyze glycosidic linkages in the fiber polysaccharides, cellulose, xylan, and glucomannan, respectively, with moderate energy input and, depending on the enzyme, sometimes with high specificity. Since the 1980s, several GH-based commercial products like ECOPULP (AB Enzymes) and FibreCare (Novozymes) have been developed to help reduce energy consumption in the processing of cellulosic fibers, which is achieved because enzymatic cleavage facilitates both internal and external fiber fibrillation.^9,10^

In addition to GHs, plant cell wall-active redox enzymes, classified as Auxiliary Activities (AAs) in the CAZy database, have gained increasing interest for application in fiber engineering.^11−15^ Of these, lytic polysaccharide monooxygenases (LPMOs; EC 1.14.99.53–56) are monocopper enzymes that hydroxylate the C1 or C4 carbon in glycosidic linkages in cellulose, resulting in oxidative cleavage of these linkages and introduction of a carboxyl (at C1) or carbonyl (at C4) group at one of the newly formed chain ends.^16^ To become catalytically active, LPMOs require the reduction of the active-site copper to Cu(I) by an electron donor (reductant).^17^ Despite an initial debate on the nature of the oxygen-containing cosubstrate, it is now generally accepted that LPMOs primarily perform a peroxygenase reaction using H_2_O_2_,^18,19^ which is several orders of magnitude faster than the monooxygenase reaction with O_2_.^20−24^ The oxidative mechanism employed by LPMOs and the consequent introduction of oxidized chain ends along the cellulose fiber broaden the possibilities for enzymatic fiber modification. Carbonyl and carboxyl groups can influence fibril interactions^25^ and serve as access points for chemical functionalization of the fiber surfaces.^14,26−29^ Furthermore, unlike cellulases, LPMOs have a flat (or only slightly grooved) substrate-binding surface, allowing them to target well-ordered, crystalline areas of cellulose.^30,31^

LPMOs have been successfully applied for cellulose processing in various areas: to improve pulp drainage and paper strength,^32,33^ to produce nanocelluloses,^14,25,34−41^ or to facilitate the dissolution of pulp fibers for regenerated cellulose products.^42−44^ However, achieving controlled, efficient LPMO-driven fiber modification at an industrial scale remains challenging due to the high enzyme concentrations and extended reaction times that are typically used. Although some studies have achieved surface modification with limited substrate degradation by optimizing reaction conditions, including substrate loading,^45,46^ enzyme dose,^14,26^ and reaction time,^46^ these studies rely on “monooxygenase” conditions, which offer limited control over the reaction. In these setups, LPMO activity is limited by the *in situ* production of H_2_O_2_ resulting from the reductant-consuming oxidase activity of the LPMO and/or abiotic oxidation of the reductant.^47−49^ Such *in situ* H_2_O_2_ production will depend on the redox properties of the LPMO and the type of reductant.^50,51^ Controlling H_2_O_2_ levels would allow control over the degree of substrate oxidation while simultaneously minimizing the risk of oxidative damage to the LPMO, which may occur if H_2_O_2_ levels are too high.^18,52^

Studies on the enzymatic saccharification of cellulose have shown that LPMOs can be controlled by managing the amount of available H_2_O_2_, either by continuous supply of H_2_O_2_ to the reaction^53^ or by its *in situ* generation, using H_2_O_2_-producing enzymes.^18,54,55^ In such reaction setups, with generally lower reductant levels, rather precise control over the extent of fiber oxidation is expected by controlling the number of catalytic cycles and by avoiding conditions (i.e., low substrate concentration and high H_2_O_2_ levels) that promote enzyme inactivation. The remaining challenges include the determination of the extent of fiber oxidation versus the generation of soluble oxidized cellulose fragments, which are known to vary with the LPMO, cellulose type, and substrate loading.^45,46,56^

In this work, we explored the concept of controlling LPMO-catalyzed fiber oxidation by sequentially supplying defined amounts of gallic acid (GA) as a reductant and H_2_O_2_ during the reaction, using fungal LPMOs that oxidize cellulose at the C1 (*Pa*AA9E), C4 (*Nc*AA9C), or both C1 and C4 carbons (*Tr*AA9A). We monitored changes in fiber properties, such as the degree of oxidation, average molecular mass, and viscosity, alongside the formation of soluble oxidized oligosaccharides for a series of LPMO reactions with varying conditions. Moreover, we assessed the correlation between the amounts of GA and H_2_O_2_ added to the reaction mixture and the amounts of LPMO-generated oxidized soluble and insoluble products.

## Materials and Methods

### Chemicals, Cellulosic Fibers, and Enzymes

Chemicals were purchased from Sigma-Aldrich-Merck. Cellulosic fibers were produced in sodium form from Whatman No. 1 filter paper (GE Healthcare; production site, China) by cold disintegration and washing processes, as described by Rahikainen et al.^57^

LPMOs *Tr*AA9A from *Trichoderma reesei* (UniProt ID, G0R6T8), *Pa*AA9E from *Podospora anserina* (UniProt ID, B2ATL7), and *Nc*AA9C from *Neurospora crassa* (UniProt ID, Q7SHI8) were produced and purified as described earlier.^46,58,59^*Trichoderma reesei* (teleomorph of *Hypocrea jecorina*) cellobiohydrolase *Tr*Cel7A (UniProt ID, G0RVK1) and *Myriococcum thermophilum* cellobiose dehydrogenase *Mt*CDH (UniProt ID, A9XK88) were produced and purified according to published protocols.^60,61^

### Oxidation of Cellulosic Fibers with LPMOs

For small-scale initial screening of controlled fiber oxidation, LPMO reactions were conducted in 200 μL of total volume of 2.5% (w/v) dry matter cellulosic fibers and 0.5 μM LPMO (*Nc*AA9C or *Pa*AA9E) in 50 mM Bis-Tris/HCl buffer, pH 7.0. As a reductant, gallic acid (GA) was added either sequentially every 15 min (15 μM for each addition, starting at *t* = 0 min) or in a single dose (1 mM) at *t* = 0 min. To some of the reactions, H_2_O_2_ (or, in the control reaction, water) was supplied at varying levels every 15 min, starting at *t* = 0 min. Reactions were incubated at 45 °C and 1000 rpm using Eppendorf Thermomixers type C (Eppendorf AG, Hamburg, Germany) for 1, 2, or 3 h. To stop the reaction, samples were kept at 99 °C for 5 min. Supernatants were filtered using 96-well filtration microplates installed on a MultiScreen vacuum manifold (Merck Millipore, Burlington, MA, USA) and stored at 4 °C before further analysis.

In subsequent experiments performed at gram scale, 0.5–1.0 g (dry weight) of cellulosic fibers were incubated with LPMO (*Pa*AA9E or *Tr*AA9A; 0.0081–0.105 μmol/g dry fiber) in 50 mM sodium phosphate buffer, pH 7.0, at 2.5% (w/v) fiber concentration in 100 mL glass bottles. The reactions were incubated at 45 °C with stirring for 3 h. In some reactions, the buffer was changed to 50 mM Bis-Tris/HCl, pH 6.5, or the temperature was reduced to 30 °C to test the impact of altering these process parameters on the efficiency of cellulose oxidation. Reactions were supplied with different amounts of GA (to reach 7.5, 15, or 30 μM) and H_2_O_2_ (to reach 25, 50, 100, or 200 μM) every 15 min, starting at *t* = 0 min. Control reactions were set up without H_2_O_2_, GA, or LPMO in each experimental series. After 3 h, the suspensions were filtered twice through a filter plate made of 60 μm mesh cloth. The resulting filtrates were stored at −20 °C until further analysis. The collected fibers were washed with Milli-Q water (100 mL) and either stored at +4 °C, for spectrophotometric analysis of aldehydes and viscosity measurements, or freeze-dried, for SEC-MALS analysis of molar mass distributions and carbonyl content.

### Analysis of Soluble LPMO Products

Quantitative analysis of oxidized soluble oligosaccharides was done using high-performance anion-exchange chromatography with pulsed amperometric detection (HPAEC-PAD) using a Dionex ICS-5000 system (Thermo Scientific, Sunnyvale, CA, USA) equipped with CarboPac PA200 analytical (3 × 250 mm) and guard (3 × 50 mm) columns, using a previously established method.^62^ The supernatants of the small-scale (200 μL) reactions were hydrolyzed with 0.45–0.9 μM *Tr*Cel7A in 50 mM Bis-Tris/HCl, pH 7.0, overnight at 37 °C, to convert soluble LPMO products to a mixture of shorter oligosaccharides. C1-oxidized standards GlcGlc1A, (Glc)_2_Glc1A, and (Glc)_3_Glc1A were produced from cellobiose, cellotriose, and cellotetraose, respectively, using *Mt*CDH, as described by Sulaeva et al.^63^ C4-oxidized standards Glc4gemGlc and Glc4gem(Glc)_2_ were produced from cellopentaose using *Nc*AA9C, as described in Müller et al.^64^

For larger-scale reactions, oxidized and native cello-oligosaccharides with a degree of polymerization of 2–4 in the supernatant were analyzed semiquantitatively using an Acquity UPLC system (Waters, Milford, MA, USA) equipped with a Hypercarb PGC column (3 μm particle size; 2.1 × 150 mm; Thermo Scientific, Waltham, MA, USA) and coupled with a Synapt G2-S mass spectrometer (Waters), as described earlier.^44^ Mass spectrometry (MS) was conducted in positive electrospray ionization (ESI) mode with traveling-wave ion mobility (TWIM) detection. The relative quantities of oligosaccharides were estimated using calibration curves made from nonoxidized cello-oligosaccharides (0.05–100 μg/mL).

### Analysis of Celluloses

Combined analysis of carbonyl groups and molar mass distribution in cellulose were carried out using a method described by Sulaeva et al.^28^ In brief, cellulose samples were labeled with the fluorescent label carbazole-9-carboxylic acid [2-(2-aminooxyethoxy)ethoxy]amide (CCOA)^65^ and then washed with deionized water. The solvent was changed to *N*,*N*-dimethylacetamide (DMAc) before dissolving the samples in DMAc/LiCl [9 % (w/v)]. The dissolved samples were then analyzed with size exclusion chromatography (SEC), using four PLgel MIXED-ALS columns in series (20 μm particle size; 7.5  ×  300 mm; Agilent Technologies, Waldbronn, Germany) connected to a multi-angle laser light scattering (MALS) detector with a diode laser (λ = 488 nm; Wyatt Dawn DSP; Wyatt Technology, Santa Barbara, CA, USA), a fluorescence detector (TSP FL2000; Thermo Separation Products, USA), and a refractive index (RI) detector (Shodex RI-71; Showa Denko K.K., Kawasaki, Japan). The data were evaluated using the Chromeleon 7, Astra 4.73, and GRAMS software packages, including calculation of the number-average (*M*_n_), weight-average (*M*_w_), and z-average (*M*_*z*_) molecular masses and the carbonyl content. Note that fibers treated with C1-oxidizing LPMOs, which introduce lactone/carboxylate groups, will be labeled at (pre-existing) reducing ends and at other, not necessarily LPMO-related oxidized, sites on the fiber, while fibers treated with C4-oxidizing LPMOs will be labeled at additional sites generated by the LPMO (two carbonyls per cleavage reaction).^28^

The intrinsic viscosity of the fiber suspensions was analyzed in duplicate according to ISO 5351-1 with a PSL Rheotek pulp viscometer equipped with capillaries PSL C, for calibration, and 2 AKV, for sample measurement (Poulten, Selfe & Lee Ltd., Burnham-on-Crouch, U.K.).

Spectrophotometric analysis of aldehydes was carried out in duplicate with Salbok’s method, in which the aldehyde groups in cellulose react with 2,3,5-triphenyl-2*H*-tetrazolium chloride,^66,67^ adopted for the analysis of LPMO-oxidized fibers.^42^

Qualitative identification of the presence of terminal aldonic acids, resulting from C1 oxidation, was performed using solution-state nuclear magnetic resonance (NMR), with previous assignments described by Koso et al.^68^ The ^1^H/^13^C 2D heteronuclear single-quantum correlation (HSQC) NMR spectrum was obtained for a *Pa*AA9E-treated fiber sample, upon sequential feeding with 15 μM GA and 100 μM H_2_O_2_, as described above.

## Results and Discussion

### Assessing LPMO-Mediated Fiber Oxidation with Sequential Addition of H_2_O_2_ Based on the Formation of Soluble Oxidized Products

LPMO action leads to the accumulation of oxidized chain ends on cellulose fibers and to solubilization of oxidized (and some native) oligosaccharides, when cellulose is cleaved near a chain end, either pre-existing or created by LPMO activity. The ratio of LPMO-generated oxidized ends in the soluble fraction generally increases over time, decreases with higher substrate concentrations, and varies by LPMO.^45,46^ At the substrate concentration used in this study, typically 50–80% of the oxidized ends end up in the soluble fraction,^45,56,69^ making the monitoring of soluble products a reliable indicator of LPMO activity.^70^

**Figure 1:**
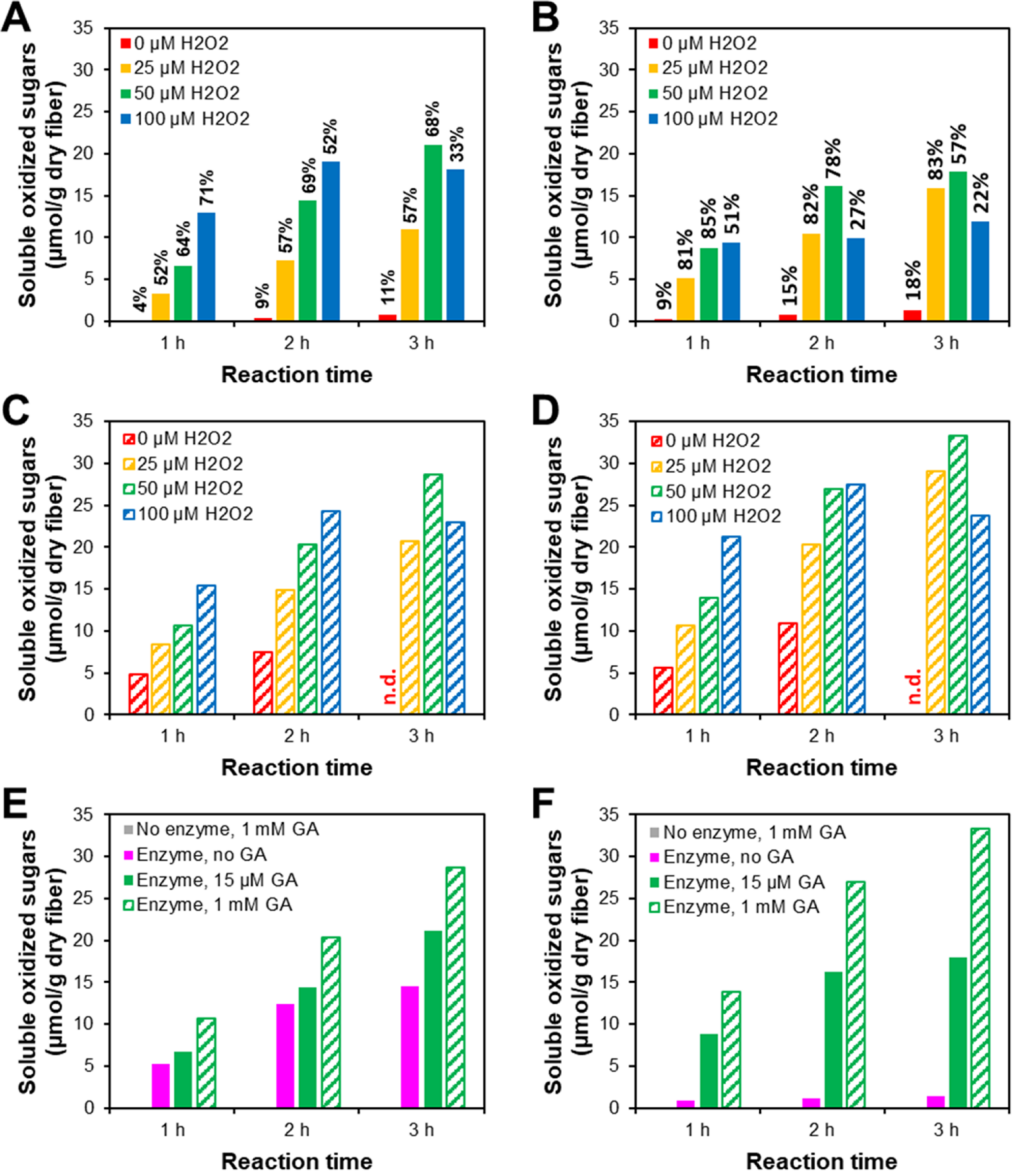
Accumulation of soluble oxidized sugars over time during treatment of Whatman No. 1 fibers with *Nc*AA9C (A, C, and E) and *Pa*AA9E (B, D, and F). Reactions (200 μL) contained 2.5% (*w*/*v*) dry matter cellulosic fibers and 0.5 μM LPMO in 50 mM Bis-Tris/HCl buffer, pH 7.0, and were incubated at 45 °C for 1, 2, or 3 h (independent reactions for each time point). In panels (A) and (B), reactions were supplemented with 15 μM GA and 25, 50, or 100 μM H_2_O_2_ every 15 min, starting at *t* = 0 min. In panels (C) and (D), reactions were supplemented with 1 mM GA in the beginning of the reaction and with 25, 50, or 100 μM H_2_O_2_ every 15 min, starting at *t* = 0 min. In the control reactions shown in panels (E) and (F), 50 μM H_2_O_2_ was added every 15 min, starting at *t* = 0 min, and reactions were set up (1) without LPMO and with a single addition of 1 mM GA, (2) with LPMO and without GA, (3) with LPMO and sequential addition of 15 μM GA every 15 min (same data as in panels (A) and (B)), and (4) with LPMO and a single addition of 1 mM GA at *t* = 0 min (same data as in panels (C) and (D)). The soluble oxidized sugars were analyzed by HPAEC-PAD. In panels (A) and (B), the percentages above the bars represent the fraction of the total amount of GA and H_2_O_2_ that is recovered as soluble oxidized products. Abbreviation: n.d., not determined. Data underlying this figure are shown in Table S1.

We monitored soluble oxidized sugars during treatment of Whatman No. 1 cellulosic fibers with two CBM-containing LPMOs, *Nc*AA9C and *Pa*AA9E, at pH 7.0 and varying GA and H_2_O_2_ levels (Figure 1). In accordance with their regioselectivity,^47,71^*Nc*AA9C generated C4-oxidized oligosaccharides and *Pa*AA9E generated C1-oxidized oligosaccharides (>98% of the detected oligosaccharides; Table S1). Sequential addition of GA and H_2_O_2_ promoted product formation by both LPMOs, with a clear correlation between the (soluble) product formation and the amount of added H_2_O_2_ at earlier time points (Figure 1A,B). Over time, enzyme inactivation became increasingly evident with both LPMOs at higher H_2_O_2_ levels, especially at 100 μM, as indicated by the plateauing of product formation. Enzyme inactivation is also apparent when looking at the fraction of the total amount of added GA and H_2_O_2_ that is recovered as soluble oxidized products (Figure 1A,B). *Pa*AA9E was more prone to redox inactivation than *Nc*AA9C. For *Pa*AA9E, product formation was severely reduced after 1 h of incubation at 100 μM H_2_O_2_ and after 2 h of incubation at 50 μM H_2_O_2_, while for *Nc*AA9C a halt in product formation was observed only after 2 h when H_2_O_2_ was added at 100 μM concentration.

In a control experiment, 1 mM GA was added in a single dose at the beginning of the reaction to ensure a high level of reductant in the reaction that keeps the LPMO in the reduced, catalytically active form, while only H_2_O_2_ was added sequentially. Compared to the sequential addition of 15 μM GA (reaching 0.18 mM after 2 h 45 min) (Figure 1A,B), these reactions resulted in higher amounts of oxidized sugars and delayed inactivation of *Pa*AA9E (Figure 1C,D), presumably because GA generates H_2_O_2_ by reacting with molecular O_2_^72^ (compare red bars in Figure 1A–D), while it can react with OH and OOH radicals and scavenge these,^73^ thereby protecting LPMOs from redox inactivation.^48^ Independent of the GA concentration, in the present reaction setups, the highest yields of soluble oxidized sugars were formed after 3 h at 50 μM H_2_O_2_ per addition (Figure 1A–D).

Control reactions without enzymes showed a negligible formation of oxidized sugars (Figure 1E,F). However, control reactions lacking GA revealed a major difference between the two LPMOs. In the absence of GA, *Nc*AA9C still produced considerable amounts of oxidized products, unlike *Pa*AA9E. This observation suggests that reducing power in the reaction mixture can reduce (activate) a fraction of the *Nc*AA9C molecules that is sufficient to productively convert the added H_2_O_2_. Further studies are needed to elucidate this phenomenon and the difference between the two LPMOs. It is well known from earlier work that the efficiency of LPMO reduction depends on both the LPMO and the reducing compound.^50^

Overall, Figure 1 not only highlights the complexity of controlling LPMO reactions but also shows that effective control is achievable with careful management of H_2_O_2_ and by preventing LPMO inactivation. Of note, LPMO inactivation further increases complexity by release of free copper, which can cause redox side reactions; although this problem is less prominent with GA, which can form a complex with free Cu(II) ions,^74^ than with, for example, ascorbic acid.^48,75^ These data support the emerging notion that cellulose-active AA9 LPMOs have widely different functionalities, including their preference for different cellulose forms,^46,56^ the ratio between soluble and insoluble products,^45,46,56^ and, as shown in Figure 1, their sensitivity to inactivation and reduction.

### Controlling Fiber Oxidation by Driving LPMO Action with Sequential Feeding of the Reductant and H_2_O_2_

**Figure 2:**
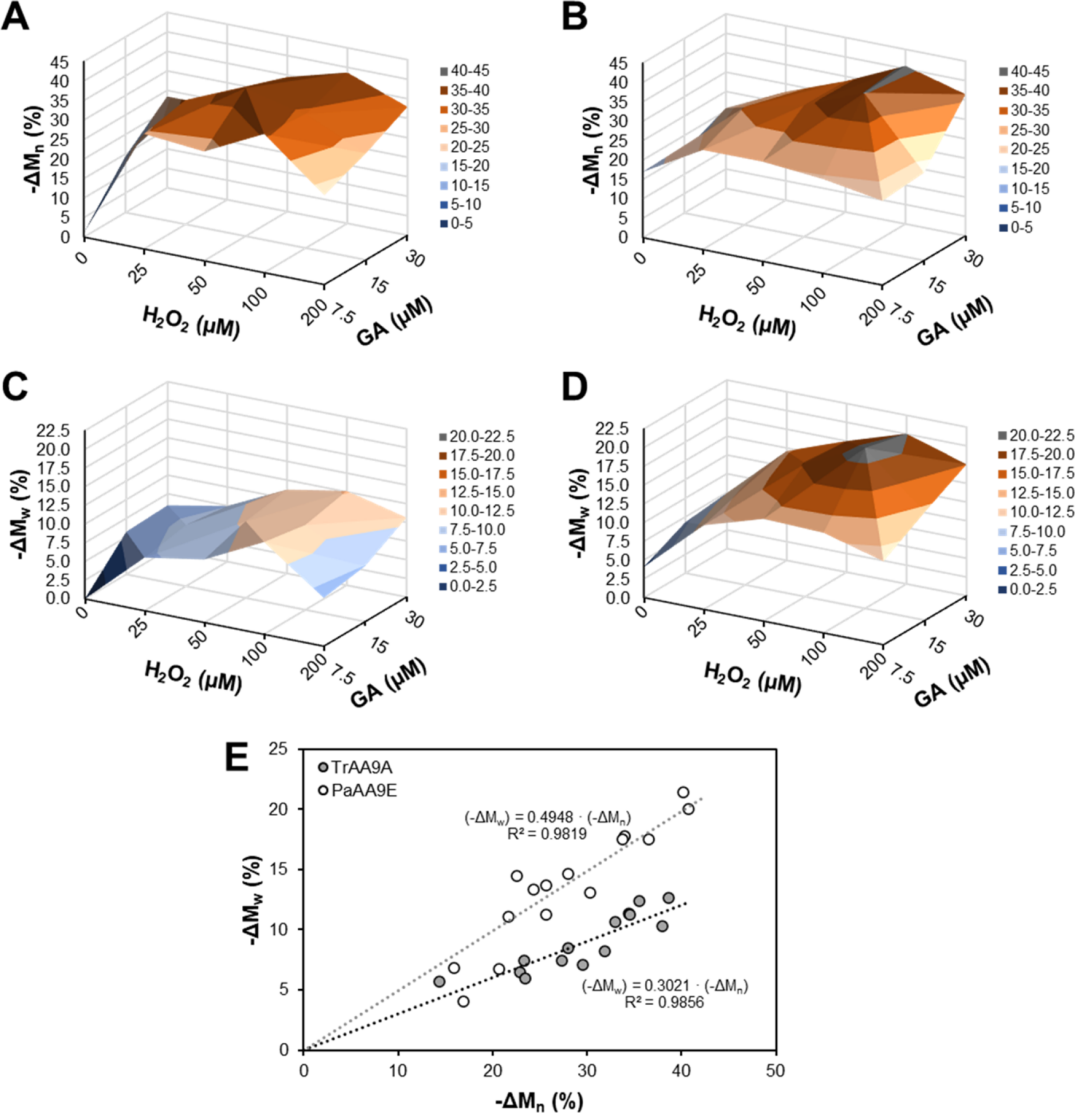
Impact of *Tr*AA9A (A, C) and *Pa*AA9E (B, D) treatments on the average molecular weight of Whatman No. 1 fibers. The reductions in *M*_n_ (−Δ*M*_n_; panels (A, B)) and *M*_w_ (−Δ*M*_w_; panels (C, D)) are provided as percentages and were calculated as (*M*_untreated fibers_ – *M*_treated fibers_)/*M*_untreated fibers_ × 100% based on the data provided in Table S4. Panel (E) shows the relationship between the reduction in *M*_n_ and *M*_w_ for each of the reactions. The LPMO reactions were carried out in 50 mM sodium phosphate buffer, pH 7.0, for 3 h, with the sequential addition of GA (7.5, 15, or 30 μM) and/or H_2_O_2_ (0, 25, 50, 100, or 200 μM) every 15 min, starting at *t* = 0 min. The enzyme dosages were 0.081 μmol/g cellulose for *Tr*AA9A and 0.105 μmol/g cellulose for *Pa*AA9E, corresponding to ca. 3 mg of protein/g of dry fiber. Control reactions without LPMO or without the addition of GA and H_2_O_2_ showed no reduction in *M*_n_ or *M*_w_ (Table S4). The following data points have been reported earlier by Sulaeva et al.:^28^ untreated fiber (reference without GA and H_2_O_2_), fibers treated with *Tr*AA9A and with sequential addition of 15 or 30 μM GA alone without H_2_O_2_, or with sequential addition of 15 μM GA and 25 or 100 μM H_2_O_2_.

**Figure 3:**
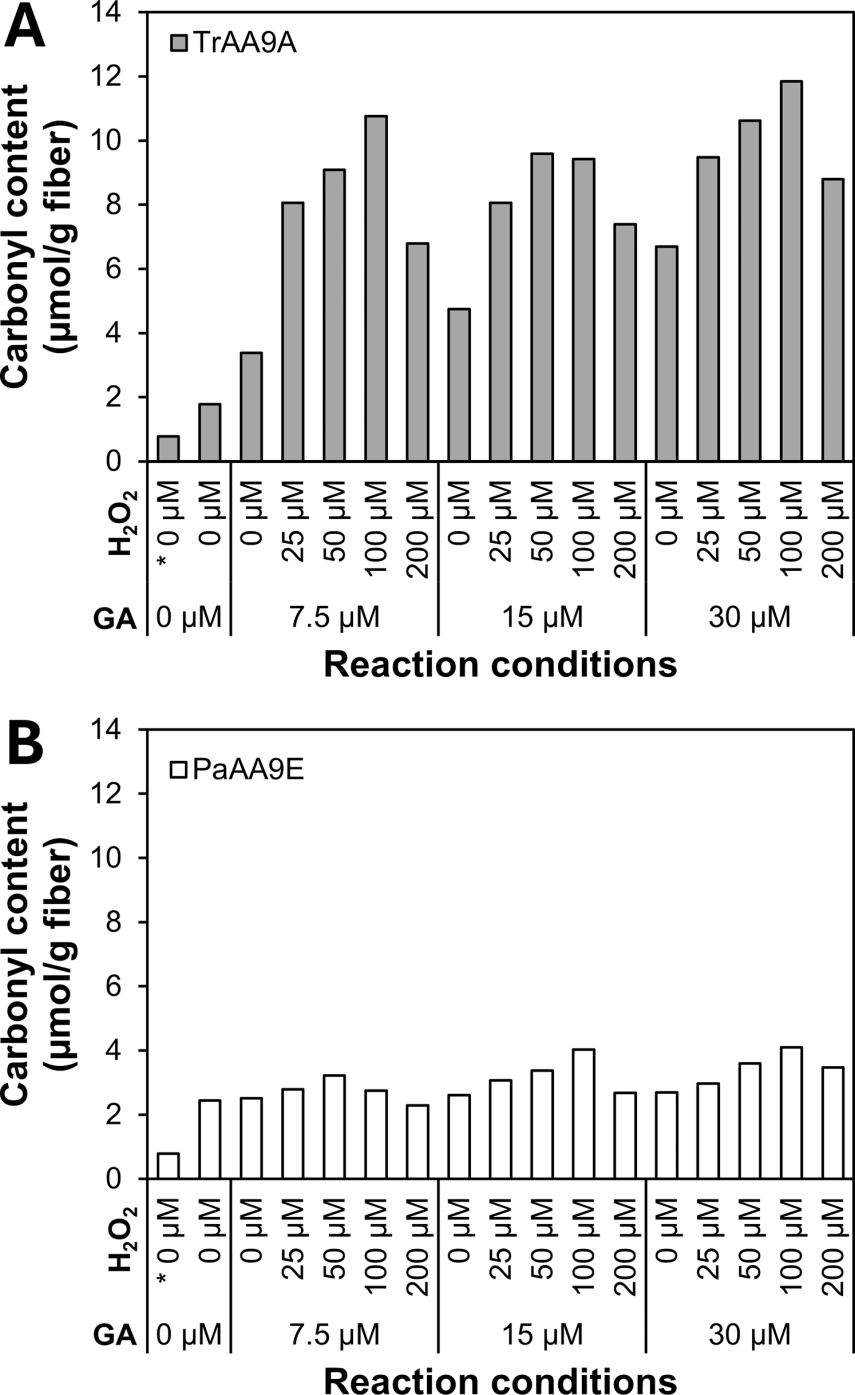
Carbonyl groups in Whatman No. 1 fibers treated with *Tr*AA9A (A) or *Pa*AA9E (B) analyzed using SEC-MALS coupled with fluorescence detection after CCOA labeling. The treatment was carried out in 50 mM sodium phosphate buffer, pH 7.0, for 3 h, with the sequential addition of GA (7.5, 15, or 30 μM) and H_2_O_2_ (0, 25, 50, 100, or 200 μM) every 15 min, starting at *t* = 0 min. The enzyme dosages were 0.081 μmol/g cellulose for *Tr*AA9A and 0.105 μmol/g cellulose for *Pa*AA9E, corresponding to ca. 3 mg protein/g dry fiber. Control reactions were set up without GA (“0 μM”) and H_2_O_2_ (“0 μM”); the asterisk indicates that the enzyme was omitted from the reaction. The following reactions were reported earlier in Sulaeva et al.:^28^ controls (0 μM GA and H_2_O_2_), reactions with *Tr*AA9A and sequential addition of 15 or 30 μM GA without H_2_O_2_, or sequential addition of 15 μM GA with 50 or 100 μM H_2_O_2_. Underlying data are listed in Table S4. Note that the detected carbonyl groups include both reducing-end aldehydes and 4-keto groups at the nonreducing end formed upon C4 oxidation.

Preliminary experiments by Sulaeva et al.^28^ found that supplying increasing amounts of GA and H_2_O_2_ during modification of Whatman No. 1 fibers with *Tr*AA9A leads to higher carbonyl content and lower-average-molecular weight. Building on these observations, we studied controlled modification of cellulose fibers using C1/C4-oxidizing *Tr*AA9A^76^ and C1-oxidizing *Pa*AA9E^71^ with varying amounts of GA and H_2_O_2_. To produce sufficient LPMO-oxidized fibers for further analysis, we performed the reactions at gram scale and in phosphate buffer, a buffer commonly used in industrial enzyme processes. We monitored the changes in fiber properties, including the molecular-weight distribution using SEC-MALS,^28,65^ intrinsic viscosity using a viscometer,^59^ and oxidation level. C4 oxidation, which leads to the formation of new reducing-end aldehydes and 4-keto groups at the nonreducing ends, was assessed by fluorescent labeling of keto groups (including 4-keto groups and reducing-end aldehydes) followed by SEC-MALS with fluorescence detection^28^ or by the spectrophotometric Salbok assay, which reacts with reducing-end aldehydes.^42,44,59,67^ C1 oxidation, yielding carboxyl groups, was analyzed qualitatively using NMR. The data, shown in Figures 2 and 3 (and detailed in Tables S2–S4), are discussed below.

Depolymerization of cellulose, indicated by decreased number-average (*M*_n_) and weight-average (*M*_w_) molecular weights, is an expected consequence of oxidative cleavage by LPMOs and has been reported in previous studies.^25,37,39,43^ Here, using the gradual addition of reductant and H_2_O_2_ to the reactions, we saw a correlation between cellulose depolymerization and the amounts of reductant and H_2_O_2_. We found that higher levels of GA and H_2_O_2_ generally increased the level of depolymerization at lower H_2_O_2_ levels (Figure 2), with the greatest reductions in *M*_n_ and *M*_w_ occurring at 15–30 μM GA and 50–100 μM H_2_O_2_ levels. In line with the experiments addressing soluble product formation discussed above, at higher H_2_O_2_ levels (200 μM), enzyme inactivation occurred, leading to lesser reductions in *M*_n_ and *M*_w_, although higher GA levels (30 μM) offered some protection against such an inactivation. These results underscore the importance of avoiding excessive H_2_O_2_ levels during LPMO treatments to ensure enzyme longevity and maximal efficiency.

Overall, in this experimental setup, enzymatic oxidation with both enzymes resulted in up to 40% reduction in *M*_n_, and such a reduction was promoted by H_2_O_2_ (Figure 2A,B). The results reveal two clear differences between the LPMOs. First, *Tr*AA9A hardly reduced the polymer length at the lowest GA levels and no added H_2_O_2_, while *Pa*AA9E was effective even under these conditions (Figure 2C,D). This suggests that the enzymes may differ in their ability to oxidize GA and generate H_2_O_2_. Additionally, reactions generated similar numbers of chain scissions (Figure 2A,B, Table S4), and *Pa*AA9E caused a clearly greater reduction in *M*_w_ compared to *Tr*AA9A (Figure 2C–E). This difference suggests more random cleavage within the fiber by *Pa*AA9E, causing a lower dispersity. It is plausible that the action of *Tr*AA9A is rather limited to the fiber surface, thereby preserving longer cellulose chains inside of the fibers. Relatively small effects of *Tr*AA9A on *M*_w_ have also been observed earlier with softwood kraft fibers.^44^

In accordance with the observed cleavage of cellulose chains, CCOA labeling revealed a notable increase in the carbonyl content of *Tr*AA9A-oxidized cellulose fibers at increasing reductant (7.5 to 30 μM) or H_2_O_2_ concentrations (25 to 100 μM), while the highest H_2_O_2_ dosage (200 μM) led to reduced carbonyl content (Figure 3A), confirming that excessive H_2_O_2_ levels disrupt LPMO activity. Irrespective of enzyme inactivation, the carbonyl content was proportional to decreases in *M*_n_ and *M*_w_ (Figure S1A,B), and the production of soluble LPMO products followed a similar trend (Table S2 and Figure 3A). Furthermore, the analysis of soluble oligosaccharides confirmed, in accordance with earlier studies,^28,46,77^ that C1/C4-oxidizing *Tr*AA9A predominantly performs C4 oxidation. The increase in carbonyl content for the reactions with *Tr*AA9A correlated well with the number of chain scissions (S_n_) calculated from *M*_n_ (*R*^2^ = 0.810; Figure S2). Notably, the carbonyl content exceeded the theoretical oxidation level, with about 2.5 rather than the expected 2.0 carbonyls per chain scission, indicating the occurrence of nonspecific fiber oxidation that does not lead to chain scission, in accordance with previous observations.^28^ For benchmarking, we performed the spectrophotometric Salbok assay for determining the reducing-end aldehyde content in *Tr*AA9A-treated cellulose fibers. While there was a fairly good correlation with the SEC-MALS data after CCOA labeling (*R*^2^ = 0.875: Figure S3), indicating that the spectrophotometric TTC assay is suitable for monitoring relative changes in the extent of C4 oxidation by LPMOs, this assay tended to systematically overestimate the amount of reducing ends. This overestimation is partly due to background signals from the untreated pulp (see the open circle in Figure S3) and primarily results from the assay protocol, where boiling the pulp samples in an alkaline solution can induce cleavages and thus create new chain ends and aldehyde groups in the cellulose chains.^78,79^

Treatment with *Pa*AA9E, a strictly C1-oxidizing LPMO,^71^ yielded predominantly C1-oxidized soluble oligosaccharides (Table S3) and minimal changes in the carbonyl content of the fibers (Figures 3B and S2), consistent with the regioselectivity of *Pa*AA9E. The slight correlation between H_2_O_2_ supply and the (still low) level of carbonyl groups in the fiber fraction (Figure 3B) is likely due to nonenzymatic redox reactions causing fiber oxidation.^28^ C1 oxidation in the fiber fraction was confirmed by NMR for samples treated with *Pa*AA9E and sequential addition of 15 μM GA and 100 μM H_2_O_2_ (Figure S4). The 2D HSQC spectrum shows the clear presence of aldonic acids (Figure S4A); however, the signal abundance of the characteristic 3_A_ position in the ^1^H NMR spectrum (Figure S4B) is not significant enough to allow for quantitation. Intrinsic viscosity measurements of *Pa*AA9E-treated fibers, on the other hand, showed a good correlation with the average molecular weight (*R* = 0.902 for *M*_n_ and *R* = 0.874 for *M*_w_; Figure S5), indicating the suitability of intrinsic viscosity for evaluating oxidative fiber degradation by this enzyme in subsequent studies.

### Effect of Enzyme Dosage, Buffer Type, and Temperature on LPMO-Driven Fiber Oxidation with Sequential Addition of GA and H_2_O_2_

**Figure 4:**
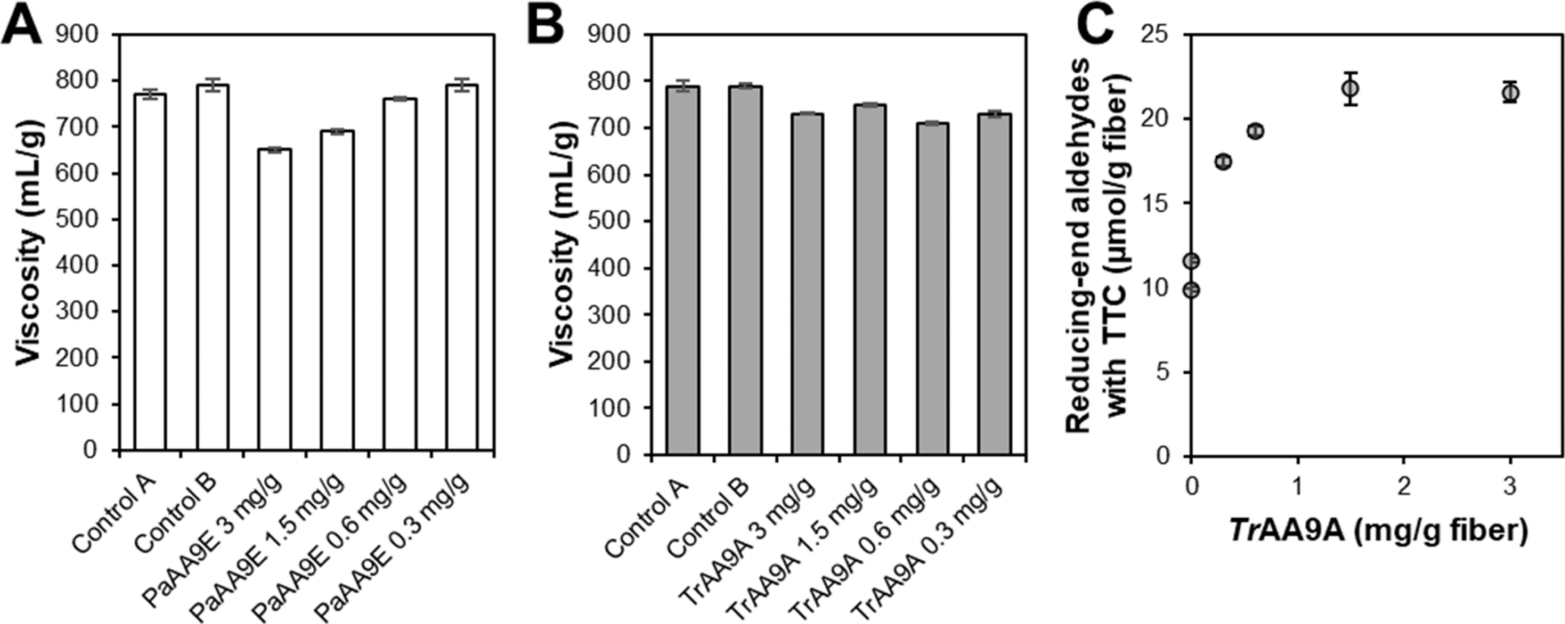
Effect of enzyme dosage on oxidation of Whatman No. 1 fibers by *Pa*AA9E (A) and *Tr*AA9A (B, C) with sequential addition of GA (15 μM) and H_2_O_2_ (50 μM in panel (A); 100 μM in panels (B) and (C)) every 15 min. Reactions were performed in 50 mM sodium phosphate buffer, pH 7.0, at 45 °C for 3 h. For the reactions with *Tr*AA9A, the reducing-end aldehydes were analyzed using Salbok’s assay (C). Control reactions were set up with LPMO and without GA or H_2_O_2_ (Control A) or with GA and H_2_O_2_ and without enzyme (Control B). The values are averages of duplicate measurements, with error bars indicating standard deviation.

We explored the impact of process parameters on fiber properties in reactions that were set up based on the data shown in Figures 2 and 3: cellulosic fibers were treated with *Tr*AA9A using 15 μM GA and 50 μM H_2_O_2_, or with *Pa*AA9E using 15 μM GA and 100 μM H_2_O_2_. The tested enzyme loadings (0.3–3.0 mg protein/g dry fiber) were in the lower end of the range of enzyme loadings tested in previous fiber oxidation studies (1–200 mg protein/g fiber),^14,25−27,38−40,46,69,80^ all of which, notably, employed “monooxygenase conditions” (i.e., reductant-driven LPMO reactions). Lowering the enzyme dosage (from 3.0 to 0.3 mg of protein/g of dry fiber) led to an enzyme dose-dependent decrease in the intrinsic viscosity of *Pa*AA9A (Figure 4A). In contrast, with *Tr*AA9A, reducing the enzyme dosage had little effect on the intrinsic viscosity (Figure 4B), though the lower enzyme load resulted in a lower increase in the reducing-end aldehyde content, as quantified by the Salbok assay (Figure 4C). The relatively smaller effect of *Tr*AA9A on fiber viscosity aligns with the fact that lower H_2_O_2_ doses were used and the observation that *Tr*AA9A treatment leads to a lesser reduction in *M*_w_ compared to *Pa*AA9E despite yielding fibers with similar *M*_n_ values (Figure 2). The viscosity data underscore the different modes of action of *Tr*AA9A and *Pa*AA9E.

Interestingly, the amount of aldehyde groups introduced by *Tr*AA9A, under the conditions used, showed a limited dose dependency (Figure 4C). Halving the enzyme load of *Tr*AA9A still yielded similar oxidation levels, and a 90% reduction in the initial enzyme load led to only 50% lower amounts of LPMO-derived oxidized chain ends. The lack of dose dependency for *Tr*AA9A suggests that, under the studied reaction conditions, the degree of fiber oxidation was primarily governed by the amount of reductant and H_2_O_2_, rather than being limited by the enzyme amount. It is conceivable that the greater dose dependency of *Pa*AA9E is due to its higher susceptibility to inactivation, as also suggested by the comparison with *Nc*AA9C described above. These observations underscore the potential to optimize enzyme usage and thereby substantially reduce process costs in LPMO-catalyzed fiber oxidation.

We also assessed the impact of varying buffer systems (50 mM Bis-Tris/HCl, pH 6.5 or 50 mM sodium phosphate, pH 7.0) and reaction temperatures (30 or 45 °C). Overall, these experiments did not yield overarching trends applying to both LPMOs, and effects were generally small (Figure S6). For individual LPMOs, the data did show tendencies that varied between the two enzymes. *Tr*AA9A formed less reducing-end aldehydes at 30 °C than at 45 °C, especially in the pH 6.5 buffer, while *Pa*AA9E reduced the viscosity to a slightly lesser extent in the pH 6.5 buffer, regardless of the temperature.

### Assessing the Potential of Controlled LPMO-Catalyzed Fiber Oxidation

**Figure 5:**
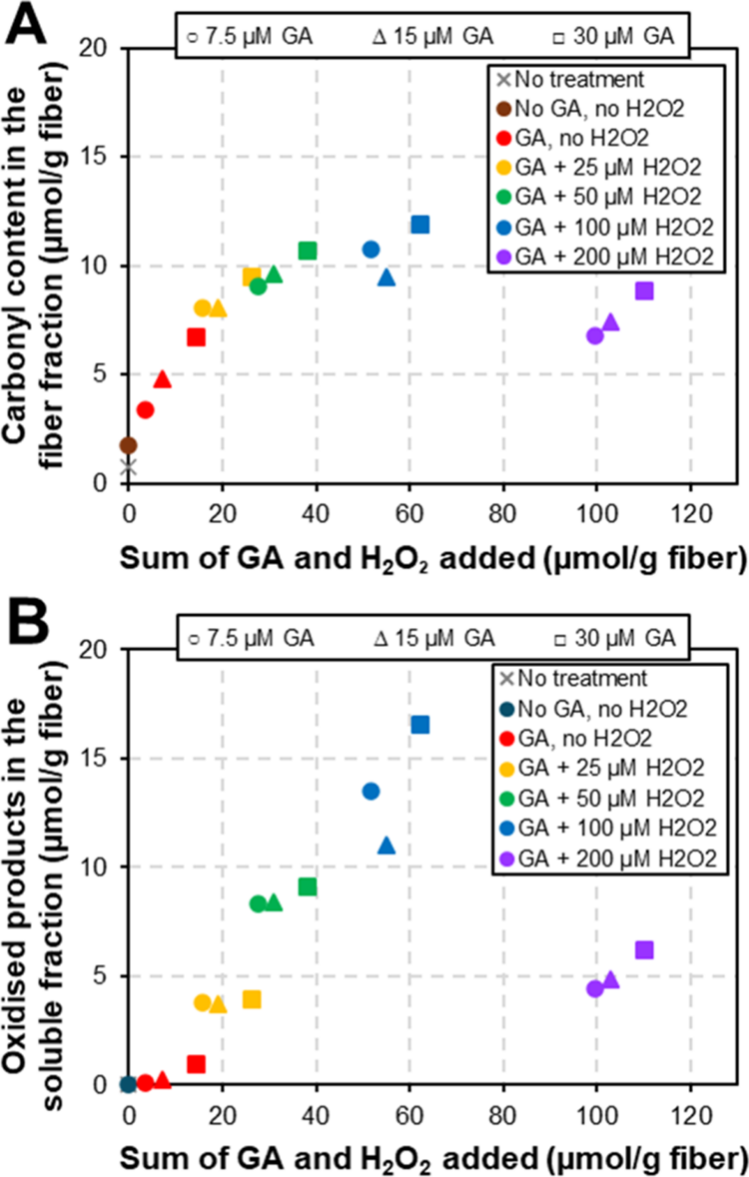
Evaluation of the efficiency of controlled fiber oxidation with *Tr*AA9A. The figure shows the carbonyl content of the fiber fraction (A) and oxidized groups in the soluble fraction (B) as the function of the sum of the reducing agent and H_2_O_2_ supplied to the reaction. The reaction conditions are the same as those indicated in Figure 2. Whatman No. 1 fibers were treated with LPMO only (brown bullet) or with LPMO and sequential addition of GA [with the concentration of 7.5 (circles), 15 (triangles), or 30 μM (squares)], alone (red symbols), or with concomitant addition of H_2_O_2_ [with the concentration of 25 (orange symbols), 50 (green symbols), 100 (blue symbols), or 200 μM (purple symbols)]. Additions were done every 15 min, and the total reaction time was 3 h. Values for the control fiber without treatment are shown as gray crosses. Note that not all of these symbols are shown in the legends that are included in the figure. The carbonyl contents in panel (A) were determined using SEC-MALS with fluorescence detection after CCOA labeling. The soluble oxidized products in panel (B) were determined using UPLC–ESI-TWIM-MS. Underlying data are given in Tables S2 and S4. Note that panel (B) only shows a proxy for total solubilization since not all products were detected and quantified (see Table S2).

A closer look at the data after the *Tr*AA9A treatment reveals interesting trends. At low GA and H_2_O_2_ levels (summed up to less than 20 μmol/g fiber), the carbonyl content in LPMO-treated fibers was high (Figure 3A; Table S4) and is proportional to the sum of the reductant and H_2_O_2_ added, while beyond this threshold, the carbonyl content starts to level off (Figure 5A). As the fiber oxidation slows down, so does the production of soluble oxidized oligosaccharides (Table S2), and it starts and continues to increase linearly with the GA and H_2_O_2_ supply in the 14–60 μmol/g fiber range (Figure 5B).

**Figure 6:**
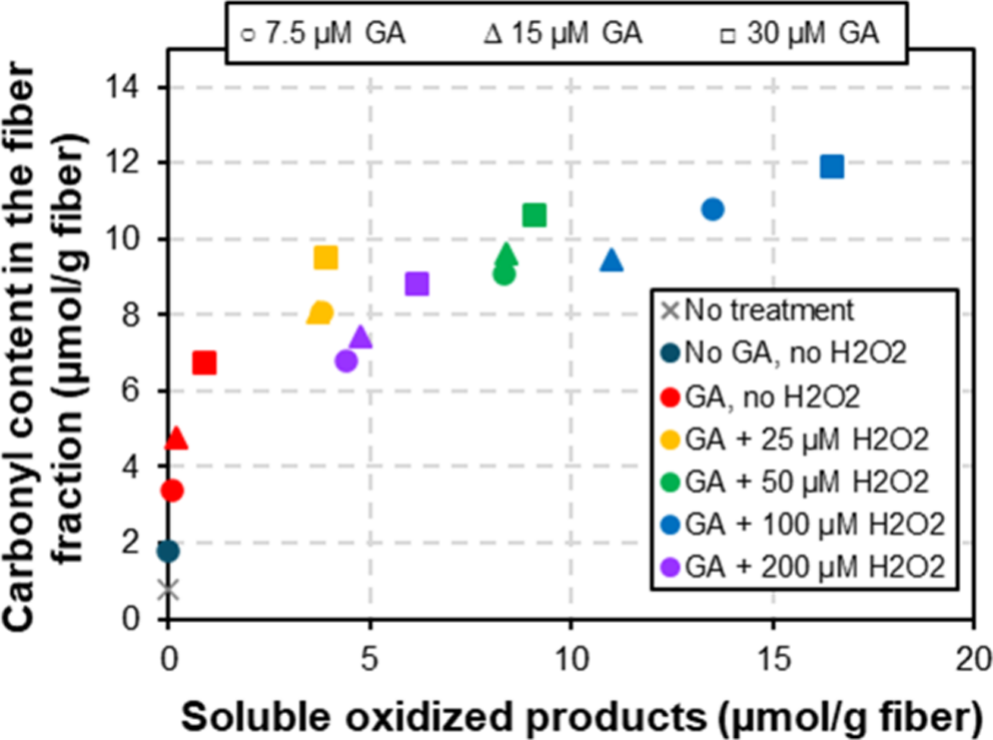
Relationship between *Tr*AA9A-generated functional groups in the fiber and the generation of soluble products. The figure shows the carbonyl content of LPMO-treated fibers as a function of the total amount of oxidized groups detected in the soluble fraction after fiber treatment. The reaction conditions are the same as those indicated in Figures 2 and 5. Whatman No. 1 fibers were treated with LPMO only (brown circle) or with LPMO along with sequential addition of GA [with the concentration of 7.5 (circles), 15 (triangles), or 30 μM (squares)], alone (red symbols), or with concomitant addition of H_2_O_2_ [with the concentration of 25 (orange symbols), 50 (green symbols), 100 (blue symbols), or 200 μM (purple symbols)]. Note that not all of these symbols are shown in the legend that is included in the figure. The value for the control fiber without treatment is shown as gray cross. Underlying data are provided in Tables S2 and S4.

Direct comparison of fiber-bound and soluble oxidized groups corroborates that initial oxidation, at the lowest amounts of GA and H_2_O_2_, primarily occurs on the fibers, with the solubilization of oligosaccharides becoming noticeable and gradually more prominent as the reaction proceeds (Figures 5 and 6). This is not unexpected, since, as the reaction proceeds, the chances of LPMOs cutting near existing chain ends increase, leading to the formation of soluble products, gradual erosion of the fiber surface, and eventual exposure of new LPMO binding sites in the underlying fibers. Previous studies^27,45,46^ have indicated that CBM-containing LPMOs promote this erosion, since the CBM anchors the LPMO to the substrate, thus increasing the likelihood of multiple cleavages occurring in close proximity. Faster catalysis in the presence of H_2_O_2_ could further contribute to an increased number of cleavages around such an “anchoring point”. The initial delay in the release of soluble products, while oxidized groups accumulate in the fibers, is in accordance with previous studies in which similar delays have been observed.^45,56^ Interestingly, Sun et al.^56^ have reported that the ratio of oxidized soluble products to fiber oxidations varies with the cellulose type,^56^ which could reflect the differences in the available surface area of cellulose^56^ or in the binding affinities^27,46,81^ and even binding modes^45^ of individual LPMOs for different substrate types. This variation among cellulose-active LPMOs suggests that the LPMO diversity evolved to handle various types of cellulose.

The formation of soluble sugars is considered a yield loss in fiber processing. Notably, *Pa*AA9E generated more soluble products in our setups than *Tr*AA9A while catalyzing similar numbers of chain scissions (calculated from *M*_n_; Figure S7, Table S4). The highest yield loss amounted to 0.6% (w/w) with *Tr*AA9A (Table S2), 0.7% (w/w) with *Nc*AA9C (Table S1), and 1.4–1.6% (w/w) with *Pa*AA9E (Tables S1 and S3), although the actual values may be higher since not all solubilized material was quantified in all reactions. Our study indicates that yield loss clearly depends on the choice of LPMO, the targeted level of fiber oxidation, and the corresponding extent of fiber degradation.

Overall, the feasibility of fiber oxidation depends on the fiber and product type, the choice of enzyme, and the process setup. The oxidation levels reached with the present optimized H_2_O_2_-driven reactions are similar to those previously observed for reductant-driven reactions with C4-oxidizing LPMOs (typically 10–20 μmol/g^28,46,82^). Reported oxidation levels for reactions with C1-oxidizing LPMOs vary from less than 1 μmol/g to more than 100 μmol/g^14,26,40,69,80^. LPMO-catalyzed oxidation, while generally achieving lower overall oxidation levels than nonenzymatic processes such as TEMPO oxidation,^14,83,84^ offers precise control (e.g., through regulated cosubstrate addition, as shown in this study) and beneficial effects on fiber properties,^14,25,32−40^ making LPMOs valuable for developing advanced cellulose materials. So far, fiber oxidation with LPMOs has been achieved using “monooxygenase” conditions, which entails using millimolar quantities of reductant and incubation overnight or for 1–2 days.^14,25,37^ In such setups, the reaction rate is governed by *in situ* H_2_O_2_ production, which depends on the redox properties of the LPMO and the type of reductant.^47−49^ Furthermore, at the time of sampling, product accumulation will likely have plateaued (partly due to enzyme inactivation),^45,46,56^ leading to overestimation of the effective reaction time. These factors make fiber oxidation with “monooxygenase” conditions costly, while the high levels of reductant can impair fiber quality (color and brightness), especially with GA. While such “monooxygenase” conditions likely could be optimized further, H_2_O_2_-driven fiber oxidation offers a more efficient alternative, with reduced doses of enzyme and GA, a reaction driven by H_2_O_2_ rather than a (more expensive) reductant, and a reaction time limited to a few hours. Importantly, our data indicate that when using conditions where enzyme inactivation is avoided, the amount of GA and H_2_O_2_ can direct the LPMO treatment to reach desired fiber modifications. Considering enzyme properties, fiber oxidation is influenced not only by LPMO regioselectivity and domain structure^45,46,56^ but also by its sensitivity to H_2_O_2_ and enzyme inactivation and impact on fiber properties like dispersity (*M*_w_/*M*_n_; this study). The fact that redox stability and binding preferences are both LPMO- and substrate-specific^46,52^ further complicates the prediction of oxidation efficiency for distinct LPMO–substrate combinations, which requires evaluation on a case-by-case basis.

## Supporting Information Available

Soluble LPMO products detected in LPMO reactions controlled with varying reductant and H_2_O_2_ levels, analysis of the LPMO-treated fibers for some of these reactions, the relationship between *M*_n_ or *M*_w_ and the carbonyl content in LPMO-treated fibers, the relationship between the carbonyl content and chain scissions in LPMO-treated fibers, the relationship between carbonyl groups quantified after CCOA labeling and reducing-end aldehydes quantified spectrophotometrically for *Tr*AA9A-treated fibers, confirmation of C1 oxidation in a *Pa*AA9E-treated fiber sample with NMR, the relationship between *M*_n_ or *M*_w_ and the intrinsic viscosity for *Pa*AA9E-treated fibers, the effect of buffer and temperature on fiber properties after LPMO treatment, and the relationship between chain scissions in the fiber fraction and oxidized products in the soluble fraction upon fiber treatment with *Tr*AA9A or *Pa*AA9E (PDF)

## Abbreviations

AA: auxiliary activity
CBM: carbohydrate-binding module
CCOA: carbazole-9-carboxylic acid [2-(2-aminooxyethoxy)ethoxy]amide
DMAc: *N*,*N*-dimethylacetamide
ESI: electrospray ionization
GA: gallic acid
GH: glycoside hydrolase
HPAEC: high-performance anion-exchange chromatography
HSQC: heteronuclear single-quantum correlation
LPMO: lytic polysaccharide monooxygenase
MALS: multi-angle laser light scattering
MS: mass spectrometry
PAD: pulsed amperometric detection
RI: refractive index
SEC: size exclusion chromatography
TEMPO: 2,2,6,6-tetramethylpiperidine-1-oxyl
TTC: 2,3,5-triphenyl-2*H*-tetrazolium chloride
TWIM: traveling-wave ion mobility
