# Supplementary material for: LPMO-Catalyzed Oxidation of Cellulosic Fibers with Controlled Addition of a Reductant and H2O2
Source: ACS Sustain Chem Eng. 2024 Dec 30;13(1):220–31. doi: 10.1021/acssuschemeng.4c06802 (PMC11734124; doi:10.1021/acssuschemeng.4c06802)
Supplement: Supplementary file 1 — sc4c06802_si_001.pdf [file sc4c06802_si_001.pdf]

Supplementary Information for

LPMO-catalyzed oxidation of cellulosic fibers with controlled addition of reductant and H_2O_2

Kaisa Marjamaa[†], Jenni Rahikainen[†,#], Fredrik G. Støpamo[‡,#], Irina Sulaeva[§], Waltteri Hosia[†], Natalia Maiorova[†], Alistair W. T. King[†], Antje Potthast[§], Kristiina Kruus[†,||], Vincent G. H. Eijsink[‡], Anikó Várnai[‡,]*

[†] VTT Technical Research Centre of Finland, P.O. Box 1000, Espoo, FI-02044 VTT, Finland

[‡] Norwegian University of Life Sciences (NMBU), Faculty of Chemistry, Biotechnology and Food Science, Chr. Magnus Falsens vei 18, Ås, 1433, Norway

[§] University of Natural Resources and Life Sciences (BOKU), Konrad Lorenz-Straße 24, Tulln an der Donau, A-3430, Austria

[||] Aalto University, P.O. Box 16100, Espoo, 00076 AALTO, Finland

[*] Corresponding author: Anikó Várnai, aniko.varnai@nmbu.no

[†] *J.R. and F.G.S. contributed equally to the manuscript.*

This PDF file (15 pages) includes:

Tables S1–S4

Figs. S1–S7

Table S1. Release of soluble oxidized products during treatment of Whatman No. 1 fibers with *Nc*AA9A or *Pa*AA9E with addition of gallic acid (GA) and H_2O_2 in various regimes. Reaction supernatants were treated with *Tr*Cel7A to convert oxidize oligomers to five different shorter oxidized species that were quantified. The values presented have been corrected for the dilution of the reaction mixture that happened as a consequence of adding various volumes of GA and H_2O_2. The asterisk indicates that only a single addition was done at the beginning of the reaction. In all other cases, the indicated amounts of gallic acid and H_2O_2 were added every 15 min starting at *t*=0. Reaction conditions are the same as in the reactions reported in **Fig. 1**. For each reaction set up, three timepoints were analyzed, and each timepoint represents an independent reaction.

**Enzyme**	**GA (μM)**	**H_2O_2 (μM)**	**Time (h)**	**GlcGlc1A (μM)**	**(Glc)$_2$Glc1A (μM)**	**(Glc)$_3$Glc1A (μM)**	**Glc4gemGlc (μM)**	**Glc4gem(Glc)$_2$ (μM)**
*Nc*AA9C	0	0	3	0.02	0.01	0.03	0.00	0.00
		50	1	0.17	0.34	0.29	101	30.4
			2	0.54	0.85	0.94	232	77.0
			3	0.81	0.98	1.1	282	79.2
	15	0	1	0.01	0.01	0.00	1.84	0.61
			2	0.06	0.05	0.09	7.89	2.32
			3	0.09	0.08	0.09	15.4	3.82
		25	1	0.16	0.22	0.19	63.6	19.2
			2	0.31	0.49	0.47	138	42.5
			3	0.57	0.62	0.68	214	59.9
		50	1	0.33	0.41	0.45	127	38.2
			2	0.76	0.98	1.1	270	88.6
			3	1.2	1.3	1.5	405	119
		100	1	0.71	0.83	1.0	244	78.0
			2	1.2	1.4	1.7	355	117
			3	1.3	1.2	1.6	358	91.0
	1000*	0	1	0.25	0.23	0.33	89.4	30.7
			2	0.38	0.54	0.58	135	48.4
		25	1	0.39	0.53	0.63	153	53.5
			2	0.87	1.0	1.3	266	103
			3	1.2	1.4	1.7	380	131
		50	1	0.70	0.73	0.92	198	66.9
			2	1.3	1.5	1.9	368	135
			3	1.9	1.9	2.6	530	181
		100	1	1.1	1.1	1.4	285	97.0
			2	1.8	1.8	2.4	445	154
			3	1.9	1.6	2.2	447	122
*Pa*AA9C	0	0	3	0.09	0.02	0.13	0.00	0.00
		50	1	10.1	1.69	10.2	0.00	0.00
			2	12.7	2.47	13.3	0.00	0.00
			3	16.5	3.46	15.9	0.00	0.00
	15	0	1	2.44	0.62	2.36	0.00	0.00
			2	6.94	2.56	8.68	0.00	0.00
			3	12.1	5.48	15.0	0.00	0.00
		25	1	51.1	18.5	58.9	0.74	0.10
			2	97.3	44.2	118	1.8	0.17
			3	155	63.3	176	3.8	0.28
		50	1	84.6	34.6	98.7	1.8	0.12
			2	154	66.7	180	4.3	0.34
			3	177	73.0	193	4.7	0.28
		100	1	92.7	36.4	104	2.2	0.15
			2	96.7	39.0	110	2.2	0.18
			3	123	47.6	125	2.9	0.17

Table S1. continued

**Enzyme**	**GA (μM)**	**H_2O_2 (μM)**	**Time (h)**	**GlcGlc1A (μM)**	**$(Glc)_2Glc1A$ (μM)**	**$(Glc)_3Glc1A$ (μM)**	**Glc4gemGlc (μM)**	**Glc4gem$(Glc)_2$ (μM)**
*Pa*AA9C	1000*	0	1	47.4	26.3	65.3	0.47	0.00
			2	93.6	47.8	131	1.6	0.12
		25	1	98.7	45.5	119	2.9	0.18
			2	189	87.6	226	4.9	0.32
			3	283	120	316	8.1	0.48
		50	1	134	59.3	151	3.0	0.25
			2	261	110	293	7.9	0.39
			3	335	135	351	11	0.73
		100	1	214	85.5	225	6.3	0.46
			2	277	114	287	8.8	0.53
			3	246	101	237	11	0.43
None	1000*	50	1	0.00	0.00	0.00	0.00	0.00
			2	0.07	0.04	0.08	0.00	0.00
			3	0.08	0.05	0.11	0.00	0.00

Table S2. Concentrations of oligosaccharides (DP2–4; µmol/g) released after treatment of Whatman No. 1 fibers with *Tr*AA9A with sequential addition of gallic acid (GA) and hydrogen peroxide every 15 min. The concentrations added are indicated in columns GA and H_2O_2. Glc2–4 refer to the length of the oligosaccharide. C1, C4, and D refer to the C1 aldonic acid, the C4 ketone/gemdiol, and the double-oxidized species, respectively. The reactions were carried out in 50 mM sodium phosphate buffer at pH 7.0 for 3 h and the soluble oxidized sugars were analyzed with UPLC-ESI-TWIM-MS. Oligosaccharide concentrations are presented as numerical values in each cell. Note that these values are only semi-quantitative and should be considered rough estimates that can show trends in oligosaccharide release when varying reaction conditions.

GA (µM)	H_2O_2 (µM)	Glc2 C1 (µmol/g)	Glc2 C4 (µmol/g)	Glc2 D (µmol/g)	Glc2 (µmol/g)	Glc3 C1 (µmol/g)	Glc3 C4 (µmol/g)	Glc3 D (µmol/g)	Glc3 (µmol/g)	Glc4 C1 (µmol/g)	Glc4 C4 (µmol/g)	Glc4 D (µmol/g)	Glc4 (µmol/g)
7.5	0	0.0	0.1	0.0	0.0	0.0	0.0	0.0	0.0	0.0	0.0	0.0	0.0
	25	0.3	1.2	0.2	0.3	0.0	0.3	0.1	0.5	0.1	0.5	0.0	0.2
	50	0.9	2.6	0.5	0.6	0.0	0.6	0.1	1.2	0.1	0.7	0.0	0.4
	100	2.4	0.7	1.0	1.1	0.0	1.1	0.2	2.6	0.3	1.8	0.0	0.9
	200	0.7	0.7	0.1	0.5	0.1	0.2	0.0	1.1	0.1	0.2	0.0	0.1
15	0	0.0	0.1	0.0	0.0	0.0	0.0	0.0	0.0	0.0	0.1	0.0	0.0
	25	0.2	1.3	0.2	0.3	0.0	0.3	0.5	0.0	0.0	0.5	0.0	0.3
	50	0.7	3.0	0.6	0.6	0.0	0.6	1.0	0.1	0.1	0.8	0.0	0.4
	100	1.0	4.4	0.5	0.9	0.0	0.6	1.6	0.2	0.2	0.6	0.0	0.4
	200	0.6	0.8	0.1	0.7	0.1	0.2	1.3	0.1	0.1	0.2	0.0	0.1
30	0	0.1	0.2	0.0	0.1	0.0	0.1	0.2	0.0	0.0	0.1	0.0	0.1
	25	0.2	1.0	0.2	0.3	0.0	0.3	0.8	0.1	0.1	0.3	0.0	0.2
	50	0.8	2.2	0.6	0.7	0.0	0.6	1.7	0.2	0.2	0.7	0.0	0.4
	100	1.8	4.4	1.3	1.1	0.1	0.9	2.7	0.4	0.4	0.9	0.0	0.5
	200	0.8	0.8	0.2	0.8	0.1	0.3	1.7	0.2	0.2	0.2	0.0	0.2

Table S3. Concentrations of oligosaccharides (DP2–4; µmol/g fiber) released after treatment of Whatman No. 1 fibers with *Pa*AA9E with sequential addition of gallic acid (GA) and hydrogen peroxide every 15 min. The concentrations added are indicated in columns GA and H_2O_2. Glc2–4 refer to the length of the oligosaccharide. C1, C4 and D refer to the C1 aldonic acid, the C4 ketone/gemdiol, and the double-oxidized species, respectively. The reactions were carried out in 50 mM sodium phosphate buffer at pH 7.0 for 3 h and the soluble oxidized sugars were analyzed with UPLC-ESI-TWIM-MS. Oligosaccharide concentrations are presented as numerical values in each cell. Note that these values are only semi-quantitative and should be considered rough estimates that can show trends in oligosaccharide release when varying reaction conditions.

GA (µM)	H_2O_2 (µM)	Glc2 C1 (µmol/g)	Glc2 C4 (µmol/g)	Glc2 D (µmol/g)	Glc2 (µmol/g)	Glc3 C1 (µmol/g)	Glc3 C4 (µmol/g)	Glc3 D (µmol/g)	Glc3 (µmol/g)	Glc4 C1 (µmol/g)	Glc4 C4 (µmol/g)	Glc4 D (µmol/g)	Glc4 (µmol/g)
7.5	0	0.2	0.0	0.0	0.0	0.1	0.0	0.0	0.0	0.2	0.0	0.0	0.0
	25	6.7	0.0	0.6	0.0	6.2	0.0	0.0	0.1	4.4	0.0	0.2	0.0
	50	7.5	0.0	1.1	0.1	7.3	0.1	0.0	0.4	4.7	0.0	0.3	0.0
	100	6.6	0.0	0.6	0.0	5.9	0.1	0.0	0.3	4.2	0.0	0.2	0.0
	200	2.2	0.0	0.3	0.0	3.0	0.0	0.0	0.2	2.8	0.0	0.1	0.0
15	0	1.0	0.0	0.2	0.0	2.6	0.0	0.0	0.0	2.6	0.0	0.1	0.0
	50	8.0	0.0	0.7	0.1	7.9	0.1	0.0	0.5	5.0	0.0	0.3	0.0
	100	8.6	0.1	1.1	0.2	8.4	0.4	0.0	3.2	5.2	0.0	0.4	0.0
	200	7.6	0.0	0.3	0.1	6.1	0.1	0.0	0.4	4.3	0.0	0.1	0.0
30	0	0.0	0.0	0.0	0.0	0.0	0.0	0.0	0.0	0.0	0.0	0.0	0.0
	25	8.3	0.0	0.4	0.0	7.6	0.0	0.0	0.3	5.0	0.0	0.2	0.0
	50	8.9	0.0	0.6	0.1	8.0	0.1	0.0	0.7	5.1	0.0	0.3	0.0
	100	9.3	0.1	1.0	0.2	8.7	0.5	0.0	4.1	5.4	0.0	0.2	0.0
	200	9.0	0.0	0.5	0.2	8.4	0.4	0.0	3.6	5.3	0.0	0.2	0.0

Table S4. Characteristics of Whatman No. 1 cellulose fibers after enzymatic oxidation with LPMOs. The cellulose (2.5%, w/v) was treated with *Tr*AA9A (0.081 µmol/g dry fiber) or *Pa*AA9E (0.105 µmol/g dry fiber) for 3 h in 50 mM sodium phosphate buffer, pH 7.0, at 45 °C. The reductant (gallic acid) was added sequentially every 15 min at concentrations ranging from 7.5 to 30 µM. In some reactions, H_2O_2 was added sequentially every 15 min immediately after gallic acid, at concentrations ranging from 25 to 200 µM. The average molecular mass values (M_n, M_w, and M_z), dispersity index ($Đ = M_w/M_n$), and carbonyl content (C=O) were determined with SEC-MALS analysis after CCOA labelling[28]. Δ C=O (µmol/g) is the difference in carbonyl content between treated and untreated samples. S_n (µmol/g) is the number of chain scissions, calculated from M_n. Reducing-end aldehydes (CHO) were analyzed spectrophotometrically with Salbok's method using TTC reagent (see the main text for a discussion of the apparent discrepancy between the C=O and CHO numbers and **Fig. S3** for the correlation between the C=O and CHO values). N.d.: not determined.

**LPMO**	**GA (µM)**	**H_2O_2 (µM)**	**M_n (kDa)**	**M_w (kDa)**	**M_z (kDa)**	**Đ**	**C=O (µmol/g)**	**CHO (µmol/g)**	**Viscosity (mL/g)**	**DP_n**	**Δ C=O (µmol/g)**	**Nr. of chain scissions (S_n) (µmol/g)**
No enzyme	0	0	210.5	384.0	588.5	1.82	0.78	10	n.d.	1298		
*Tr*AA9A	0	0	210.2	396.7	609.0	1.89	1.78	9	n.d.	1296	1.00	0.01
*Tr*AA9A	7.5	0	208.3	386.2	586.2	1.85	3.38	13	n.d.	1285	2.60	0.05
*Tr*AA9A	7.5	25	148.4	356.7	565.1	2.40	8.06	19	n.d.	915	7.28	1.99
*Tr*AA9A	7.5	50	151.5	351.6	564.7	2.32	9.09	21	n.d.	934	8.31	1.85
*Tr*AA9A	7.5	100	135.7	336.6	570.8	2.37	10.76	23	n.d.	837	9.98	2.62
*Tr*AA9A	7.5	200	162.3	359.2	583.2	2.21	6.80	16	n.d.	1001	6.02	1.41
*Tr*AA9A	15	0	180.2	362.1	578.6	2.01	4.75	16	n.d.	1111	3.97	0.80
*Tr*AA9A	15	25	143.4	352.5	582.3	2.46	8.06	21	n.d.	8842	7.28	2.22
*Tr*AA9A	15	50	130.5	344.6	569.9	2.64	9.59	25	n.d.	805	8.81	2.91
*Tr*AA9A	15	100	138.1	340.4	585.3	2.33	9.43	24	n.d.	852	8.65	2.49
*Tr*AA9A	15	200	153.0	355.4	585.8	2.32	7.39	20	n.d.	944	6.61	1.79
*Tr*AA9A	30	0	161.2	361.1	583.0	2.24	6.69	17	n.d.	994	5.91	1.45
*Tr*AA9A	30	25	161.4	355.4	574.6	2.20	9.48	20	n.d.	995	8.70	1.45
*Tr*AA9A	30	50	137.9	340.9	562.0	2.47	10.62	22	n.d.	851	9.84	2.50
*Tr*AA9A	30	100	129.1	335.5	561.8	2.60	11.85	23	n.d.	796	11.07	3.00
*Tr*AA9A	30	200	141.2	343.2	573.9	2.43	8.80	18	n.d.	871	8.02	2.33

Table S4. continued

LPMO	GA (µM)	H_2O_2 (µM)	M_n (kDa)	M_w (kDa)	M_z (kDa)	Đ	C=O (µmol/g)	CHO (µmol/g)	Viscosity (mL/g)	DP_n	∆ C=O (µmol/g)	Nr. of chain scissions (S_n) (µmol/g)
*Pa*AA9E	0	0	212.6	397.3	606.1	1.87	2.44	n.d.	770	1311	1.66	0
*Pa*AA9E	7.5	0	174.8	368.3	580.9	2.11	2.51	n.d.	720	1078	1.73	0.97
*Pa*AA9E	7.5	25	156.5	340.9	558.1	2.18	2.79	n.d.	670	965	2.01	1.64
*Pa*AA9E	7.5	50	156.4	331.6	546.2	2.12	3.22	n.d.	620	965	2.44	1.64
*Pa*AA9E	7.5	100	159.3	332.7	545.8	2.09	2.74	n.d.	660	982	1.96	1.53
*Pa*AA9E	7.5	200	164.8	341.3	554.5	2.07	2.28	n.d.	680	1016	1.50	1.32
*Pa*AA9E	15	0	177.0	357.9	581.7	2.02	2.60	n.d.	710	1092	1.82	0.90
*Pa*AA9E	15	25	146.7	333.7	547.6	2.27	3.06	n.d.	650	905	2.28	2.07
*Pa*AA9E	15	50	139.1	315.8	524.8	2.27	3.37	n.d.	630	858	2.59	2.44
*Pa*AA9E	15	100	126.0	301.8	521.4	2.40	4.02	n.d.	580	777	3.24	3.19
*Pa*AA9E	15	200	162.9	328.4	526.5	2.02	2.67	n.d.	680	1005	1.89	1.39
*Pa*AA9E	30	0	166.9	358.2	578.7	2.15	2.69	n.d.	670	1029	1.91	1.24
*Pa*AA9E	30	25	151.6	327.9	547.5	2.16	2.97	n.d.	660	935	2.19	1.85
*Pa*AA9E	30	50	139.4	316.8	527.8	2.27	3.59	n.d.	640	860	2.81	2.42
*Pa*AA9E	30	100	124.7	307.0	538.5	2.46	4.10	n.d.	600	769	3.32	3.27
*Pa*AA9E	30	200	133.7	316.7	537.8	2.37	3.47	n.d.	620	825	2.69	2.73

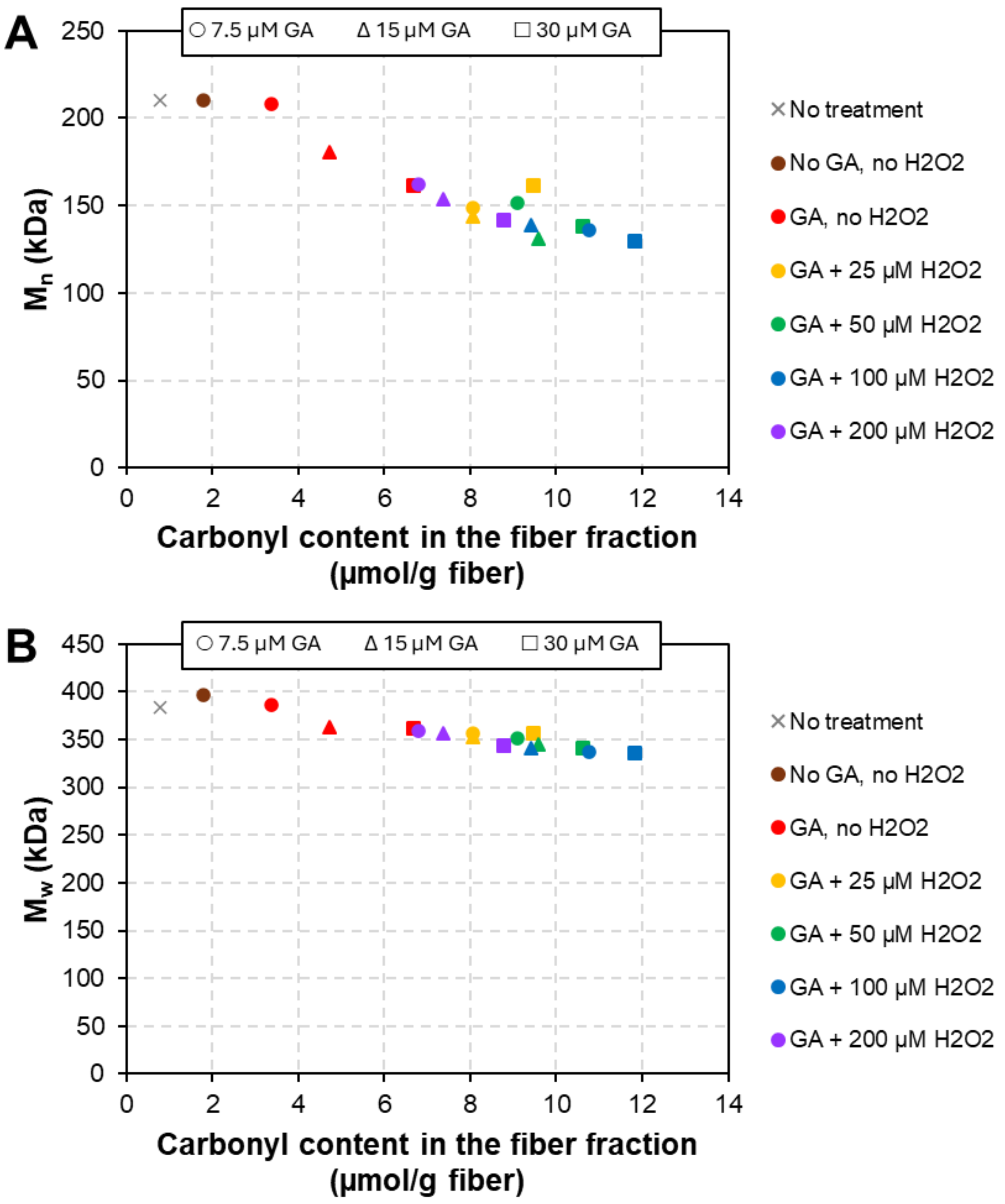

Figure S1. Relationship between the carbonyl content and M_n **(A)** or M_w **(B)** of *Tr*AA9A-treated Whatman No. 1 fibers. Cellulosic fibers were treated with LPMO only (brown circle) or with LPMO along with either a single addition of 1 mM GA without H_2O_2 (black diamond) or sequential addition of GA [in the concentration of 7.5 (circles), 15 (triangles), or 30 μM (squares)], alone (red symbols) or with H_2O_2 [in the concentration of 25 (orange symbols), 50 (green symbols), 100 (blue symbols), or 200 μM (purple symbols)]. Note that not all of these symbols are shown in the legends that are included in the figure.

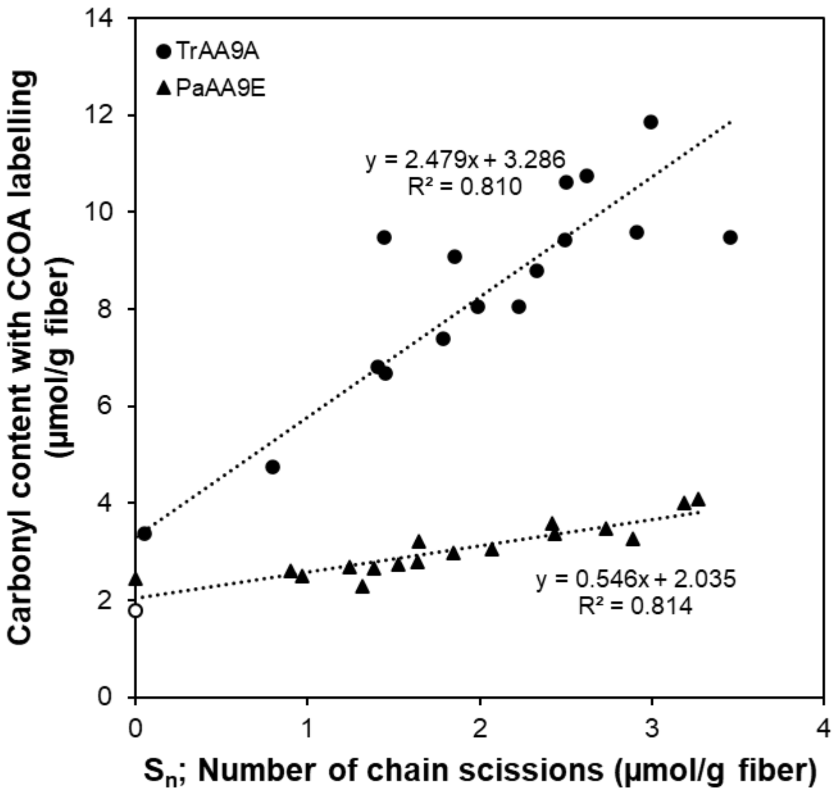

Figure S2. Relationship between carbonyl groups detected by CCOA labelling and chain scissions in the fiber fraction derived from SEC-MALS data. The samples used in the analysis include the untreated Whatman No. 1 cellulosic fibers (open symbol) and cellulosic fibers treated with 0.081 µmol/g cellulose of primarily C4-oxidizing *Tr*AA9A (filled circles) or 0.105 µmol/g cellulose of C1-oxidizing *Pa*AA9E (filled triangles) for 3 h in 50 mM sodium phosphate buffer, pH 7.0, with sequential addition of gallic acid (7.5, 15, or 30 µM), either alone or together with H_2O_2 (25, 50, 100, or 200 µM) every 15 min (data provided in **Table S4**). Note that C4 oxidation is expected to generate two carbonyls per scission (see Sulaeva et al.[28] for further discussion).

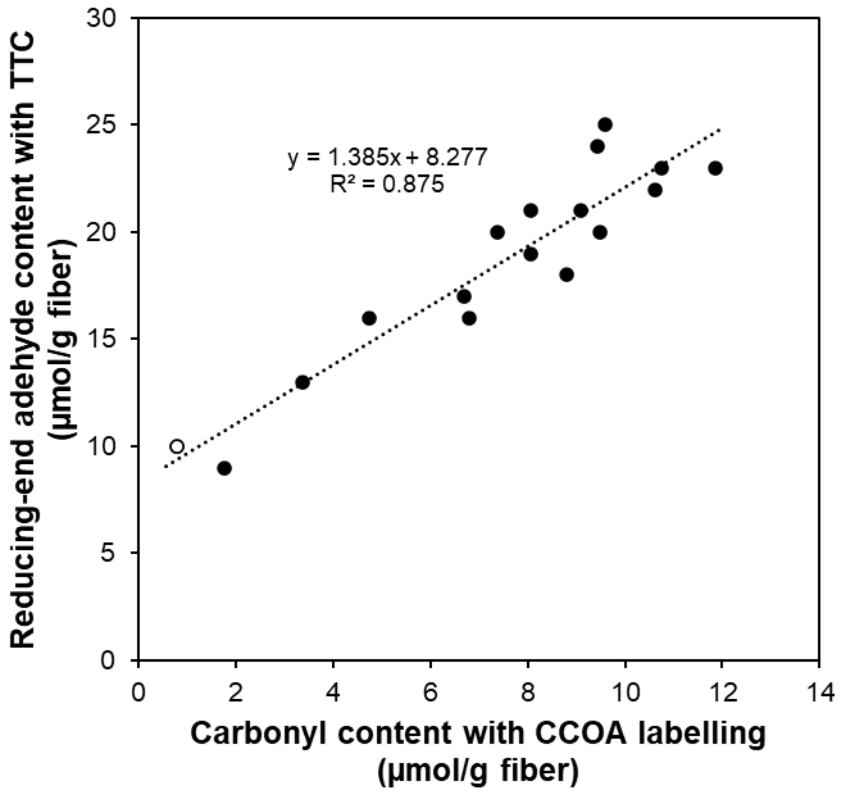

Figure S3. Relationship between carbonyl groups quantified after CCOA labelling and reducing-end aldehydes quantified with the spectrophotometric Salbok's assay using 2,3,5-triphenyl-2H-tetrazolium chloride (TTC). The samples used in the analysis include the untreated Whatman No. 1 cellulosic fibers (open symbol) and cellulosic fibers treated with *Tr*AA9A (0.081 µmol/g cellulose) for 3 h in 50 mM sodium phosphate buffer, pH 7.0, with sequential addition of gallic acid (7.5, 15, or 30 µM), either alone or together with H_2O_2 (25, 50, 100, or 200 µM) every 15 min (data provided in **Table S4**).

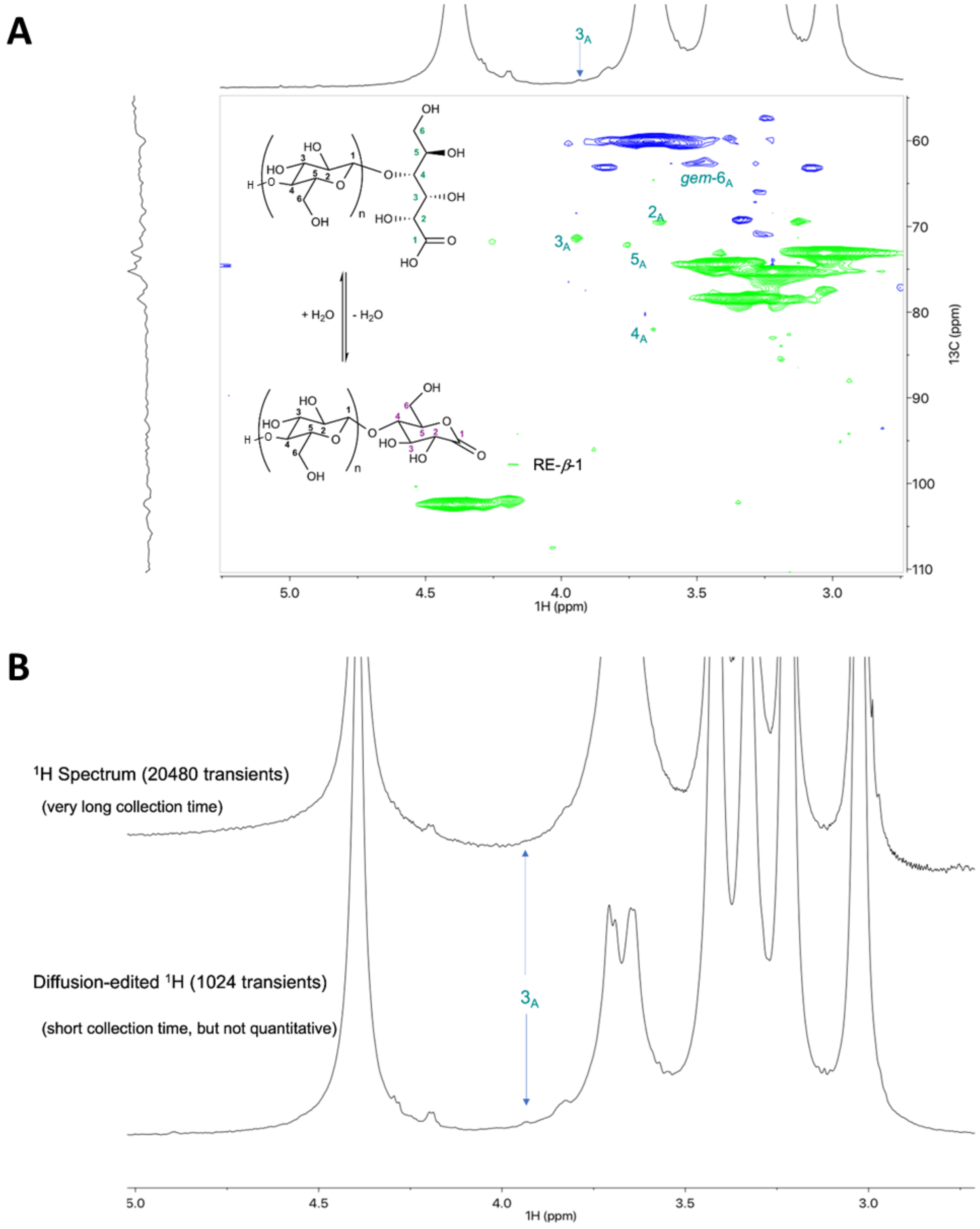

Figure S4. (A) 2D HSQC NMR spectrum of cellulosic fibers treated with *Pa*AA9E-treated fibers and sequential addition of 15 µM GA and 100 µM H_2O_2. The spectrum shows the clear presence of correlations corresponding to the open-chain 2,3,4,5 & *geminal*-6 positions (2_A to *gem*-6_A) for the previously assigned terminal aldonic acid functionality, resulting from oxidation at the C1 position. **(B)** ^{1}H NMR spectra, with the 3_A position only visible in the diffusion-edited ^{1}H spectrum.

Although the signal of the 3_A position is not overlapping with that of any main polymeric anhydroglucose unit peaks in the diffusion-edited ^{1}H NMR spectrum, its abundance is not significant enough to allow for a crude quantitation, even after extensive data collection. The enzymatic treatment was carried out with feeding of 15 µM GA and 100 µM H_2O_2 every 15 min.

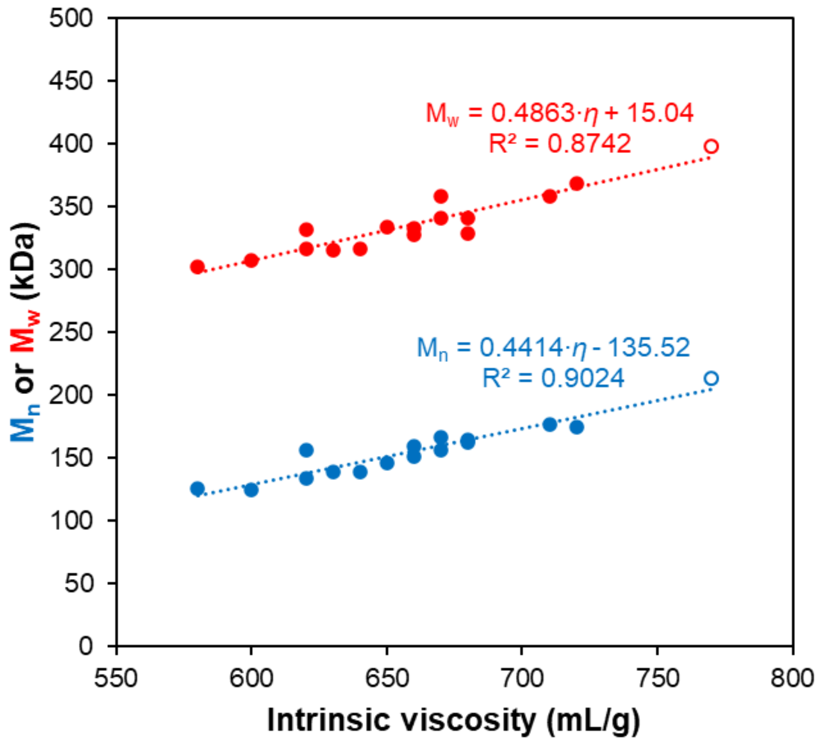

Figure S5. Relationship between the number- (M_n; blue) or weight-average (M_w; red) molecular weight and intrinsic viscosity of LPMO-treated Whatman No. 1 cellulosic fibers. The samples used in the analysis are cellulosic fibers treated with *Pa*AA9E (0.105 µmol/g cellulose) for 3 h in 50 mM sodium phosphate buffer, pH 7.0, with sequential addition of gallic acid (7.5, 15, or 30 µM), either alone or together with H_2O_2 (25, 50, 100, or 200 µM) every 15 min (data shown in **Table S4**). Data for control reactions without addition of gallic acid and H_2O_2 are shown as open symbols.

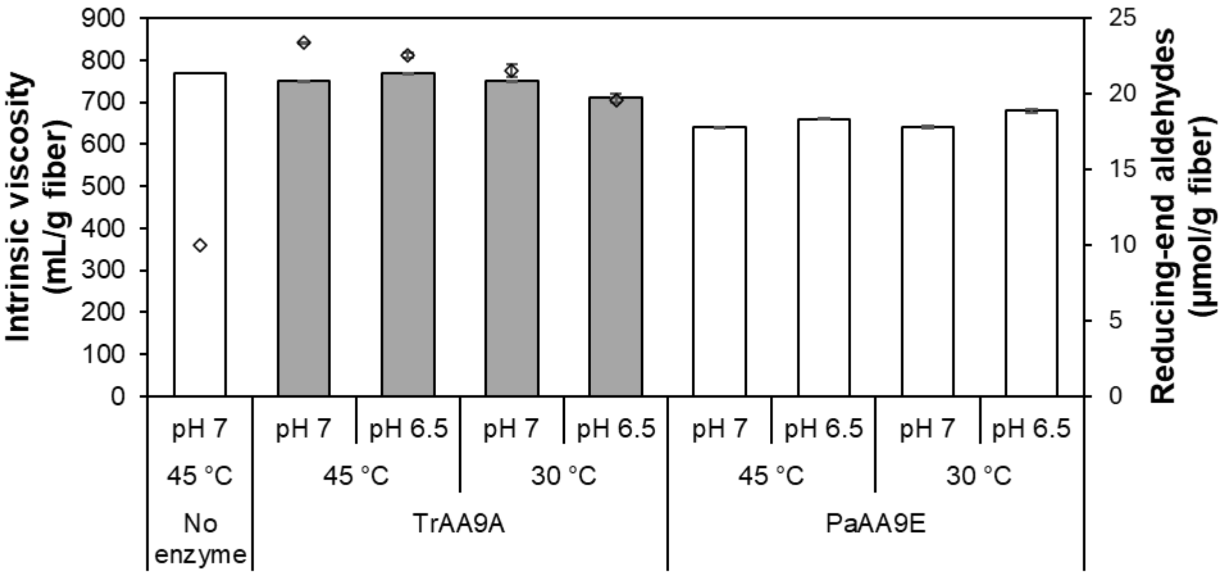

Figure S6. The effect of the buffer system and reaction temperature on oxidation of Whatman No. 1 cellulosic fibers with LPMOs *Tr*AA9A (left) and *Pa*AA9E (right). Intrinsic viscosity is shown by grey (*Tr*AA9A) and white (*Pa*AA9E) bars; the content of reducing-end aldehydes measured with the Salbok's assay is shown by diamonds (only relevant for *Tr*AA9A). Reactions (single reaction for each condition) were set up either in 50 mM Bis/Tris (pH 6.5) or in 50 mM sodium phosphate (pH 7) at 45 or 30 °C with sequential addition of gallic acid (15 µM) and H_2O_2 (50 µM for *Tr*AA9A and 100 µM for *Pa*AA9E) every 15 min. The total reaction time was 3 h. The enzyme dosages were 0.081 µmol/g dry fiber for *Tr*AA9A and 0.105 µmol/g dry fiber for *Pa*AA9E. The values are averages of duplicate measurements with error bars indicating standard deviation. Control reaction without LPMO (denoted as 'No enzyme') was carried out with the addition of gallic acid (15 µM) and H_2O_2 (50 µM) every 15 min.

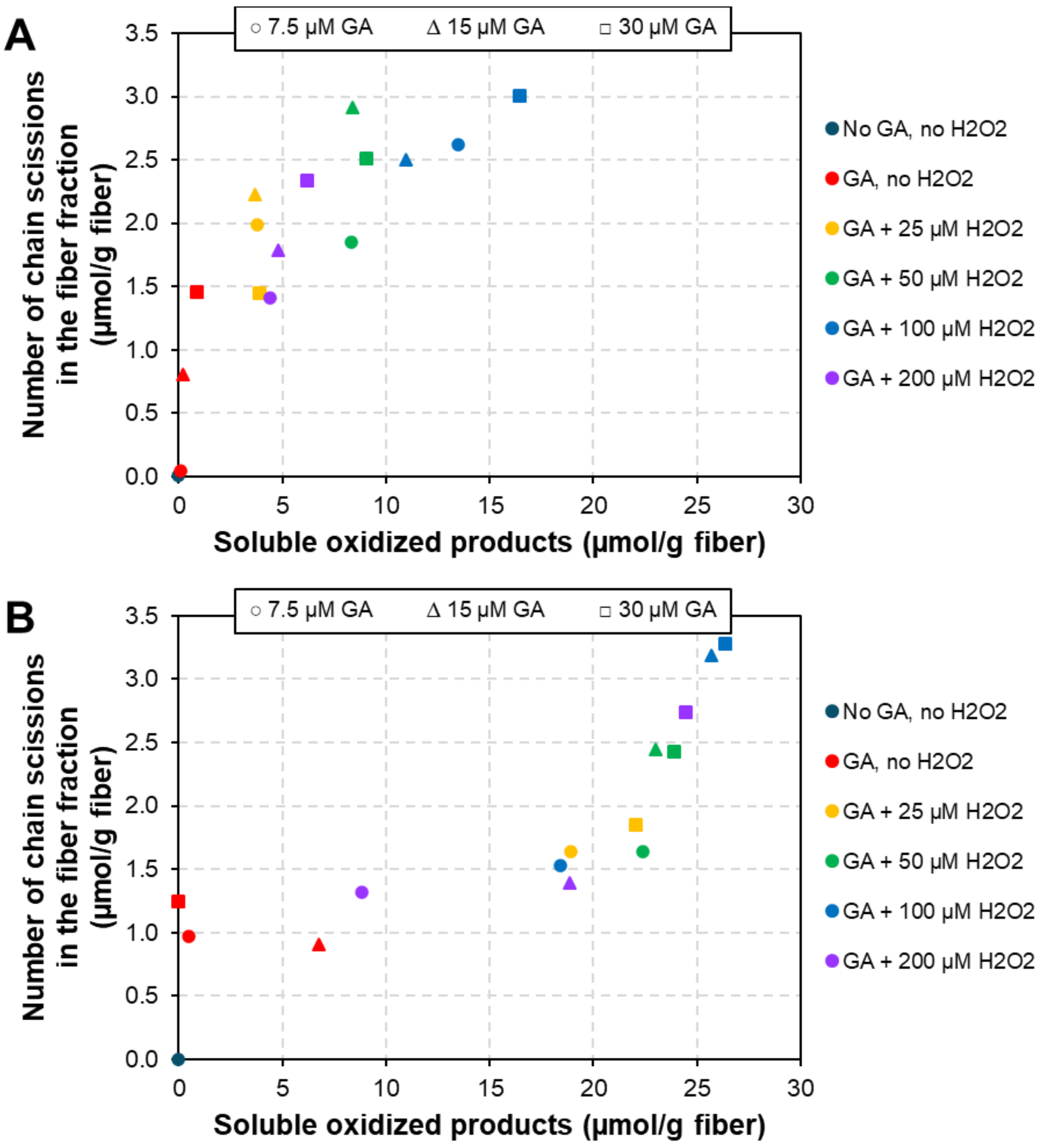

Figure S7. Relationship between chain scissions in the fiber fraction and detected oxidized products in the soluble fraction after treatment of Whatman No. 1 cellulose fibers with *Tr*AA9A **(A)** or *Pa*AA9E **(B)**. Chain scissions were calculated from the DP_n obtained with SEC-MALS; the soluble oxidized products were estimated using UPLC-ESI-TWIM-MS data. The reaction conditions are equal to those indicated in **Figs. 2, 5, and 6**. Whatman No. 1 fibers were treated with LPMO only (brown circle) or with LPMO along with sequential addition of GA [in the concentration of 7.5 (circles), 15 (triangles) or 30 µM (squares)], alone (red symbols) or with concomitant addition of H_2O_2 [in the concentration of 25 (orange symbols), 50 (green symbols), 100 (blue symbols), or 200 µM (purple symbols)]. Note that not all of these symbols are shown in the legends that are included in the figure. Underlying data are provided in **Tables S2–S4**.
